# The association of physical activity and sedentary behavior with depression in US adults: NHANES 2007–2018

**DOI:** 10.3389/fpubh.2024.1404407

**Published:** 2024-06-21

**Authors:** Yanli Meng, Ning Ma, Yixin Shi, Ning Zhang, Jing Wu, Xia Cui, Wenquan Niu

**Affiliations:** ^1^Graduate School, Beijing University of Chinese Medicine, Beijing, China; ^2^Beijing University of Chinese Medicine Third Affiliated Hospital, Beijing, China; ^3^Center for Evidence-Based Medicine, Capital Institute of Pediatrics, Beijing, China

**Keywords:** depression, physical activity, sedentary behavior, association, mediation

## Abstract

**Objectives:**

Depression is largely preventable, and strategies that can effectively suppress its development are imperative. We aimed to examine whether physical activity and sedentary behavior were associated with depression and explore the possible mediatory role of complete blood count in this association.

**Methods:**

In this cross-sectional study, data were integrated from the National Health and Nutrition Examination Study (2007–2018). Depression was defined using the Patient Health Questionnaire-9. The risk for depression, expressed as odds ratio (OR) and 95% confidence interval (CI), was quantified by survey-weighted logistic regression analyses.

**Results:**

A total of 31,204 respondents were analyzed. Significance was identified for all, except walking or bicycling per week, types of physical activity, and sedentary behavior. Per 1 standard deviation (SD) increment in metabolic equivalent of task (MET) of weekly vigorous recreational physical activity was associated with 31.3% decreased depression risk (adjusted OR: 0.687, 95% CI: 0.5663–0.840). Per 1 SD increment in sitting time can increase depression risk by 22.4% (adjusted OR: 1.224, 95% CI: 1.131–1.325). In subsidiary analyses, the association with depression was reinforced in respondents aged ≤65 years and those overweight or obese. Mediation analyses revealed significant effects for red blood cell (RBC) on total MET (19.4%) and moderate work-related physical activity (MWPA) (22.0%), and for red cell distribution wide (RCDW) on vigorous work-related physical activity (17.7%), moderate work-related physical activity (13.1%), total MET (11.2%), and sitting time (16.4%) (*p* < 0.01).

**Conclusion:**

Our findings indicate that more physical activity and less sitting time were associated with a lower likelihood of having depression among US adults, and this association was probably mediated by RBC and RCDW.

## Introduction

Depression is the leading cause of disability, and it affects approximately 7.8% of US adults (1). Statistics from the Global Burden of Disease 2019 showed that over 256 million people suffered from depressive disorders (2), reflecting an increase of 18.7% over the past decade.^1^ Depression can profoundly impact every aspect of daily life, including performance at school, productivity at work, and relationships with families and friends. More seriously, depression can precipitate self-harm and suicide (3). Because depression is largely preventable, strategies or interventions that can effectively suppress its onset or reverse its progression are imperative to reduce the mental health burden.

Evidence is mounting, indicating regular physical activity in the prevention and treatment of depression (4). Pearce et al. meta-analyzed the association between physical activity and depression and concluded that mental health can benefit significantly from regular physical activity, even at levels below the public health recommendations (5). A study from Japan reported that only vigorous physical activity and work-related exercise were positively correlated with depression (6). Despite differences in the type and intensity of physical activity, the majority of studies have supported the protective role of physical activity in the development of depression. Compared to work-related physical activity, leisure-time physical activity more easily brings a sense of joy due to one’s own preference (7, 8). As revealed by another meta-analysis (9), leisure-time physical activities with low or moderate intensity can lower the risk of incident depression, while high-intensity activities were associated with a substantiated risk of incident depression. Moreover, the type and intensity of physical activity are generally age-dependent. Zhang and colleagues have written an excellent review on physical activity and depression by demonstrating an inverse association between physical activity and depressive symptoms in older adults (7). Similar to increased physical activity, reduced time spent on sitting was also reported to be beneficial for mental health (10), and sedentary behavior is increasingly regarded as a distinct cardiovascular risk factor (11). Physical activity and sedentary behavior have become a focus of much research, especially using the National Health and Nutrition Examination Survey (NHANES) database (12–15). The association of physical activity and sedentary behavior with depression has been exhaustively investigated, with no consensus on their implications, probably due to diverse participant characteristics, unrepresentative study populations, and individually underpowered studies (16).

To address this issue and yield more information, we aimed to examine whether physical activity and sedentary behavior were associated with a significant risk of depression in a nationally representative US adult population. Meanwhile, we attempted to explore the possible mediatory role of complete blood count (CBC) in this association.

## Methods

### Study design and population

Data were extracted from the NHANES, 2007–2018, which can be accessed/downloaded at the website https://wwwn.cdc.gov/nchs/nhanes/Default.aspx. The NHANES was initiated in 1999 and comprised a series of continuous, multistage, nationally representative surveys weighted to be representative of the non-institutionalized civilian US adults and children. Survey data from in-home interviews and mobile examination centers were released in 2-year cycles. The Capital Institute of Pediatrics academic review board deemed this study exempt from ethical review and informed consent owing to the use of deidentified, publicly available data. This study was conducted according to the STROBE reporting guideline.

**Figure 1:**
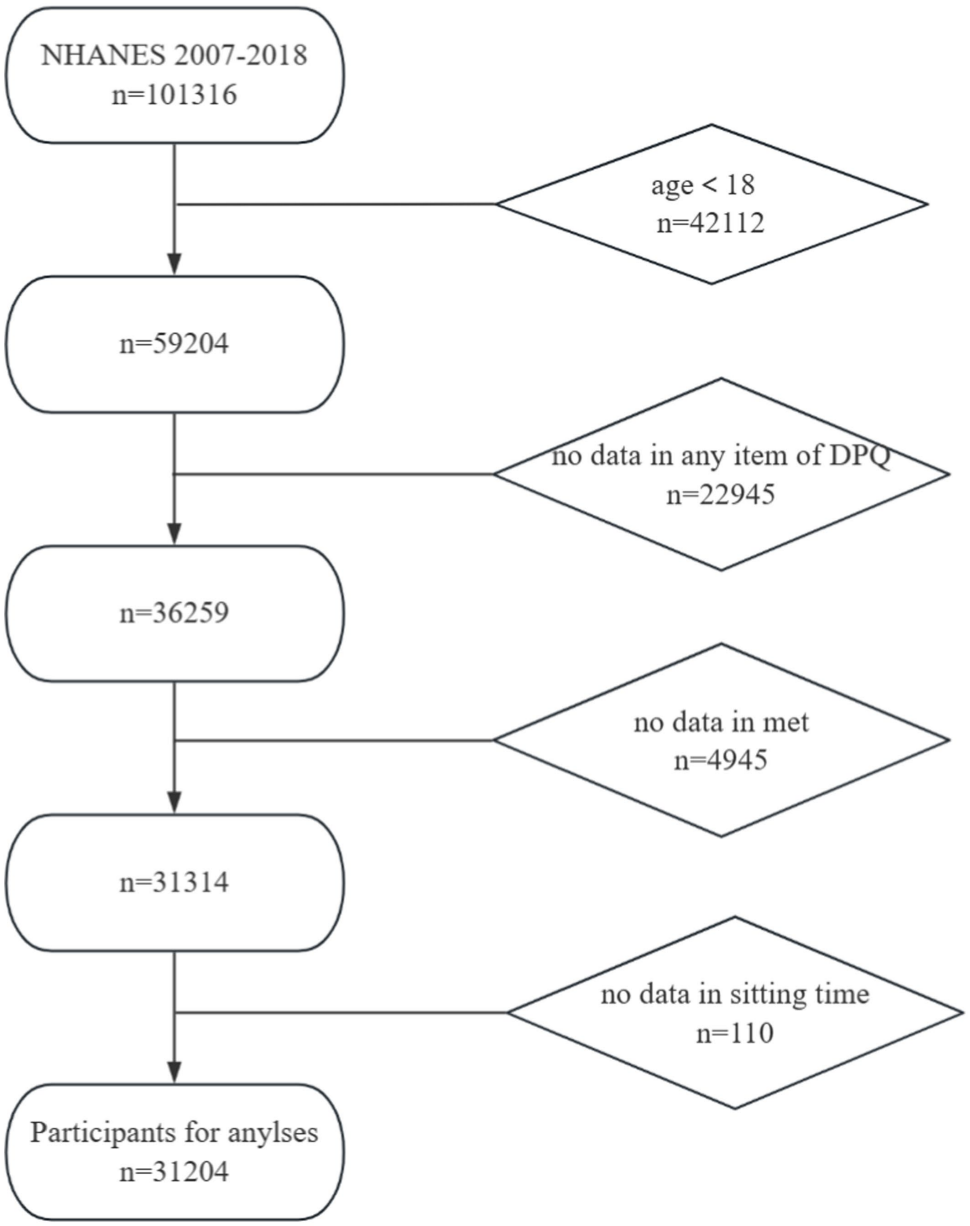
Selection process of eligible respondents in this study.

Initially, 101,316 respondents from the NHANES 2007–2018 were eligible for inclusion. After excluding 42,112 respondents who were aged <18 years, 22,945 respondents with incomplete data on the Depression Screener Questionnaire (DPQ), 4,945 respondents with incomplete data on the metabolic equivalent of task (MET), and 110 respondents without sedentary behavior information, 31,204 respondents were included in the final analysis. The selection process of study respondents is displayed in Figure 1.

### Depression definition

The 9-question Patient Health Questionnaire (PHQ-9) is the 9-item depression scale of the Patient Health Questionnaire recommended by the Diagnostic and Statistical Manual of Mental Disorders, 5th Edition (DSM-V) (17). Each item in the PHQ-9 scores from 0 to 3 (0 denotes “not at all,” 1 denotes “several days,” 2 denotes “more than half the days,” and 3 denotes “nearly every day”). The scores for all items are added to obtain a total score (range: 0 to 27). Depression is defined as 10 points and higher (18), and this cutoff point was validated elsewhere (19).

### Physical activity and sedentary behavior

Information on physical activity and sedentary behavior was collected from the Computer-Assisted Personal Interviewing System at home. Self-report instruments were used in this survey. The physical activity questionnaire (PAQ) is based on the Global Physical Activity Questionnaire (GPAQ).^2^ Specifically, the PAQ covers five types or intensities of physical activity, including vigorous work-related physical activity (VWPA) (MET: 8), moderate work-related physical activity (MWPA) (MET: 4), walking or bicycling for transportation (TPA) (MET: 4), vigorous recreational physical activity (VRPA) (MET: 8), and moderate recreational physical activity (MRPA) (MET: 4). Each type of physical activity was estimated in MET-hours per week to derive total physical activity, using the formula physical activity (MET-h/wk) = MET×weekly frequency×duration of each physical activity, as previously reported (20). In addition, the total MET per week was derived by summing the MET values of five types of physical activity.

Sedentary behavior was determined by the question, “How much time do you usually spend sitting or reclining on a typical day?”

### Covariates

As previously reported (21–26), survey cycle, age, sex, race and ethnicity, body weight and height, education, cigarette smoking, alcohol drinking, marital status, income ratio, and history of diabetes were frequently treated as covariates from the NHANES database. Additionally, medication for depression was also treated as a covariate.

The body mass index (BMI) was calculated as weight in kilograms divided by height in meters squared, and it was categorized into underweight (BMI <18.5 kg/m^2^), normal weight (18.5 kg/m^2^ ≤ BMI < 25.0 kg/m^2^), overweight (25 kg/m^2^ ≤ BMI < 30 kg/m^2^), and obesity (BMI ≥ 30.0 kg/m^2^). Education was divided into five categories: less than 9th grade, 9th–11th grade (including 12th grade with no diploma), high school graduate/GED or equivalent, some college or AA degree, and college graduate or above. The question “Have you/Has SP smoked at least 100 cigarettes in your/his/her entire life?” was used to define cigarette smoking, and the question “In any one year, have you/has SP had at least 12 drinks of any type of alcoholic beverage? By a drink, I mean a 12 oz. beer, a 4 oz. glass of wine, or an ounce of liquor” was used to define alcohol drinking. Marital status was divided into married, widowed, divorced, separated, never married, and living with a partner. Family poverty income ratio (PIR) was classified into high (PIR ≥3), middle (1 ≤ PIR <3), and low (PIR <1). Diabetes mellitus (DM) was diagnosed if one of the following criteria was met: (1) prior diagnosis by a doctor; (2) hemoglobin A1c >6.5%; (3) fasting glucose >7.0 mmol/L; (4) random blood glucose >11.1 mmol/L; (5) blood glucose of 2-h oral glucose tolerance test >11.1 mmol/L; and (6) taking anti-insulin or anti-diabetes drugs. Detailed covariate information can be found on the website http://www.cdc.gov/nchs/nhanes/.

### CBC

CBC was measured using the Beckman Coulter MAXM analyzer, based on the Beckman Coulter method of counting, sizing, and automatic diluting and mixing for sample processing according to the laboratory procedures described in the NHANES General Information for Public Files and Laboratory Files.

### Statistical analyses

Study respondents were weighted to be representative of the non-institutionalized civilian resident US population. The distributions of continuous variables were assessed for normality using the skewness and kurtosis test. Continuous variables are expressed as mean ± standard deviation (SD) in the case of normal distributions or median (95% confidence interval) in the case of skewed distributions, and categorical variables as count (95% confidence interval). Between-group comparisons were made using Student’s *t*-test and the Kruskal–Wallis equality-of-populations rank test for normally distributed and skewed continuous variables, respectively, and using the χ^2^ test or Fisher’s exact test for categorical variables.

The NHANES database can be weighted to be representative of the non-institutionalized civilian resident US population (27). As previously reported (9, 28), a survey-weighted logistic regression analysis was adopted to examine the association of physical activity and sedentary behavior with depression risk, with effect-size estimates expressed as odds ratio (OR) and 95% confidence interval (95% CI) before and after adjusting for covariates. As a prerequisite, in multivariate logistic regression analysis, respondents with missing covariate data were excluded. Specifically, three models were constructed: model 1 (without adjustment), model 2 (adjusting for survey cycle, age, sex, and race/ethnicity), and model 3 (additionally adjusting for BMI, education, drinking, smoking, marital status, income ratio, and history of diabetes). In addition to overall association, subgroup analyses were performed according to covariates on a categorical scale, and the interaction of covariates with physical activity and sedentary behavior in association with depression was also explored. Considering the limited number of respondents reporting medication for depression, sensitivity analyses were performed before and after additionally adjusting for antidepressant medication for depression, and subgroup analyses were performed with and without medication. Moreover, the possible mediatory impact of CBC on the association of physical activity and sedentary behavior with depression was explored.

All statistical analyses were performed using STATA software, version 17 (Stata Corp, College Station, TX, United States). Significance was defined if the two-sided *p*-value was less than 0.05.

## Results

### Study population

A total of 31,204 respondents (mean age: 47 [SD: 18.55] years; 15,834 women and 15,370 men) were analyzed. Table 1 summarizes the baseline characteristics of the study population according to depression; respondents were diagnosed with depression if the PHQ-9 score was ≥10. 3,830 (9.07%). The weighted mean age was 46 years for respondents with and without depression. Depression was overrepresented in women compared to men (63.57% vs. 36.43%).

**Table 1:** Baseline characteristics of study population by depression, NHANES 2007–2018.

Variables	Total	Without depression	With depression	*p*
Sample size	31,204	28,374	2,830	
Age, yrs	47 (46, 47)	46 (46, 47)	46 (45, 47)	0.235
BMI, kg/m^2^	29.05 (28.88, 29.23)	28.91 (28.73, 29.09)	30.65 (30.24, 31.06)	<0.001
Sex				<0.001
Women	51.12 (50.50, 51.73)	49.99 (49.34, 50.64)	64.08 (61.72, 66.38)	
Men	48.88 (48.27, 49.50)	50.01 (49.36, 50.66)	35.92 (33.63, 38.28)	
Race				<0.001
Non-Hispanic white	66.68 (63.84, 69.40)	67.00 (64.16, 69.72)	63.02 (59.12, 66.76)	
Non-Hispanic black	11.14 (9.76, 12.70)	10.97 (9.59, 12.50)	13.17 (11.28, 15.32)	
Mexican American	8.71 (7.33, 10.32)	8.75 (7.37, 10.35)	8.27 (6.53, 10.43)	
Others Hispanic	5.82 (4.94, 6.84)	5.62 (4.77, 6.60)	8.07 (6.46, 10.03)	
Other race	7.66 (6.89, 8.50)	7.67 (6.89, 8.54)	7.47 (6.32, 8.81)	
Education				<0.001
Less than 9th grade	4.93 (4.43, 5.49)	4.63 (4.14, 5.18)	8.35 (7.25, 9.61)	
9-11th grade	10.31 (9.51, 11.17)	9.71 (8.92, 10.58)	17.13 (15.39, 19.03)	
High school graduate	23.11 (22.03, 24.23)	22.83 (21.74, 23.95)	26.41 (24.24, 28.69)	
Some college degree	31.61 (30.61, 32.63)	31.40 (30.34, 32.49)	34.00 (31.43, 36.67)	
College graduate or above	30.03 (28.11, 32.03)	31.43 (29.48, 33.44)	14.11 (11.55, 17.13)	
Cigarette smoking				<0.001
No	56.09 (54.85, 57.32)	57.57 (56.34, 58.79)	39.17 (36.31, 42.10)	
Yes	43.91 (42.68, 45.15)	42.43 (41.21, 43.66)	60.83 (57.90, 63.69)	
Alcohol drinking				0.305
No	23.37 (21.99, 24.81)	23.29 (21.88, 24.76)	24.32 (22.27, 26.51)	
Yes	76.63 (75.19, 78.01)	76.71 (75.24, 78.12)	75.68 (73.49, 77.74)	
Marital status				<0.001
Married	54.98 (53.55, 56.41)	56.59 (55.15, 58.02)	36.59 (33.90, 39.36)	
Widowed	5.66 (5.31, 6.03)	5.42 (5.09, 5.78)	8.36 (7.09, 9.83)	
Divorced	10.35 (9.83, 10.90)	9.75 (9.21, 10.31)	17.32 (15.70, 19.07)	
Separated	2.38 (2.15, 2.64)	2.12 (1.90, 2.36)	5.43 (4.68, 6.28)	
Never married	18.36 (17.20, 19.56)	18.05 (16.84, 19.33)	21.87 (20.16, 23.67)	
Living with partner	8.26 (7.71, 8.86)	8.07 (7.50, 8.69)	10.44 (8.97, 12.13)	
Income ratio				<0.001
High income	49.30 (47.25, 51.35)	51.31 (49.29, 53.33)	26,11 (23.17, 29.28)	
Middle income	35.90 (34.42, 37.41)	35.19 (33.67, 36,75)	44.03 (41.69, 46,40)	
Low income	14.80 (13.69, 15.99)	13.50 (12.46, 14.60)	29.86 (27.25, 32,61)	
Diabetes diagnosed				0.047
No	0.27 (0.19, 0.38)	0.25 (0.18, 0.34)	0.49 (0.23, 1.05)	
Yes	99.73 (99.62, 99.80)	99.75 (99.66, 99.82)	99.51 (98.95, 99.77)	
Physical activity (MET-hour/week)				<0.001
Per-week MET of VWPA	23.66 (22.06, 25.26)	23.84 (22.19, 25.49)	21.58 (17.99, 25.17)	0.216
Per-week MET of MWPA	20.56 (19.69, 21.43)	20.68 (19.83, 21.54)	19.10 (16.74, 21.47)	0.162
Per-week MET of TPA	5.07 (4.68, 5.45)	5.06 (4.66, 5.45)	5.20 (4.43, 5.98)	0.712
Per-week MET of VRPA	4.40 (4.12, 4.67)	4.61 (4.32, 4.90)	1.96 (1.59, 2.33)	<0.001
Per-week MET of MRPA	6.44 (6.20, 6.68)	6.62 (6.37, 6.87)	4.42 (3.81, 5.03)	<0.001
TMET per week	60.12 (57.88, 62.37)	60.81 (58.49, 63.13)	52.26 (46.88, 57.64)	0.003
Sitting time (min)	372.62 (367.38, 377.86)	371.05 (465.69, 376.41)	390.61 (377.06, 404.15)	0.005
RBC (1,000 cells/μL)	4.69 (4.68, 4.70)	4.70 (4.68, 4.71)	4.64 (4.62, 4.66)	<0.001
RCDW (%)	13.24 (13.21, 13.27)	13.21 (13.19, 13.25)	13.50 (13.43, 13.57)	<0.001

NHANES, National Health and Nutrition Examination Survey; BMI, body mass index; MET, metabolic equivalent of task; VWPA, vigorous work physical activity; MWPA, moderate work physical activity; TPA, walking or bicycling for transportation; VRPA, vigorous recreational physical activity; MRPA, moderate recreational physical activity; TMET, total MET; RBC, red blood cell count; RCDW, red cell distribution width. Data are expressed as median (95% confidence interval) for continuous variables and count (95% confidence interval) for categorical variables.

### Overall analyses

Table 2 shows the association of physical activity and sedentary behavior with depression. Before adjustment, only VRPA, MRPA, TMET, and sedentary behavior were associated with a significant risk of depression. After full adjustment, significance was retained for all except TPA types of physical activity and sedentary behavior. For example, per 1 SD increment in MET of weekly VRPA was associated with a 31.3% decreased risk for depression (fully adjusted OR = 0.687, 95% CI: 0.563 to 0.840). By contrast, per 1 SD increment in sitting time can increase depression risk by 22.4% (fully adjusted OR = 1.224, 95% CI: 1.131 to 1.325).

**Table 2:** Overall association of physical activity and sedentary behavior with depression in survey-weighted logistic regression models.

Physical activity and sitting time	Model 1	Model 2	Model 3
Per-week MET of VWPA (1 SD increment)	0.965 (0.911, 1.023)	1.007 (0.951, 1.066)	0.845 (0.774, 0.922)^***^
Per-week MET of MWPA (1 SD increment)	0.960 (0.904, 1.020)	0.976 (0.919, 1.036)	0.872(0.790, 0.962)^**^
Per-week MET of TPA (1 SD increment)	1.009 (0.962, 1.059)	1.015 (0.968, 1.064)	0.985 (0.901, 1.077)
Per-week MET of VRPA (1 SD increment)	0.618 (0.536, 0.714)^***^	0.621 (0.537, 0.718)^***^	0.687 (0.563, 0.840)^***^
Per-week MET of MRPA (1 SD increment)	0.764 (0.680, 0.858)^***^	0.776 (0.689, 0.873)^***^	0.783 (0.666, 0.920)^**^
TMET per week (1 SD increment)	0.907 (0.845, 0.973)^**^	0.943 (0.879, 1.013)	0.782 (0.699, 0.875)^***^
Sitting time (1 SD increment)	1.100 (1.031, 1.174)^**^	1.115 (1.045, 1.190)^**^	1.224 (1.131, 1.325)^***^

NHANES, National Health and Nutrition Examination Survey; SD, standard deviation; MET, metabolic equivalent of task; VWPA, vigorous work physical activity; MWPA, moderate work physical activity; TPA, walking or bicycling for transportation; VRPA, vigorous recreational physical activity; MRPA, moderate recreational physical activity; TMET, total MET. Data are expressed as odds ratio (95% confidence interval). In multivariate logistic regression analysis, respondents with missing covariate data were excluded. Model 1: Only the survey cycle was adjusted. Model 2: Survey cycle, age, sex, and race/ethnicity were adjusted. Model 3: Survey cycle, age, sex, race/ethnicity, body mass index, education, drinking, smoking, marital status, income ratio, and history of diabetes were adjusted. ^*^p < 0.05, ^**^p < 0.01, ^***^p < 0.001.

### Subsidiary analyses

The association of physical activity and sedentary behavior with depression by covariates was provided in Table 3. By age, the association was reinforced for VRPA and sedentary behavior in respondents aged 18–65 years. Specifically, for VRPA, the odds ratio (OR) was 0.700 (95% CI: 0.574 to 0.852), and for sitting time, the OR was 1.215 (95% CI: 1.117 to 1.321). By sex, the impact of physical activity and sedentary behavior on depression was more obvious in men than in women regarding TMET and setting time. Per 1 SD increment in sitting time, women were 1.232 times (95% CI, 1.116 to 1.361) more likely to have depression, while men were 1.216 times (95% CI, 1.050 to 1.408) more likely to have depression.

**Table 3:** Subsidiary association of physical activities and sedentary behavior with depression by covariates.

Subgroups		Per-week MET of the following physical activity						
		VWPA	MWPA	TPA	VRPA	MRPA	TMET	Sitting Time
Age, years	(18, 65)	0.843 (0.764, 0.930)^***^	0.869 (0.780, 0.967)^*^	1.001 (0.916, 1.094)	0.700 (0.574, 0.852)^***^	0.781 (0.657, 0.928)^**^	0.784 (0.693, 0.886)^***^	1.215 (1.117, 1.321)^***^
	>65	0.934 (0.728, 1.198)	0.953 (0.750, 1.211)	0.716 (0.541, 0.947)^*^	0.420 (0.142, 1.245)	0.832 (0.495, 1.400)	0.790 (0.534, 1.169)	1.328 (1.077, 1.638)^**^
Sex	Women	0.949 (0.849, 1.062)	0.881 (0.769, 1.009)	0.963 (0.843, 1.100)	0.694 (0.515, 0.935)^*^	0.681 (0.542, 0.855)^**^	0.827 (0.703, 0.974)^*^	1.232 (1.116, 1.361)^***^
	Men	0.761 (0.661, 0.877)^***^	0.863 (0.741, 1.006)	0.994 (0.888, 1.112)	0.656 (0.490, 0.879)^**^	0.906 (0.753, 1.090)	0.735 (0.626, 0.864)^***^	1.216 (1.050, 1.408)^**^
BMI, kg/m^2^	<18.5	2.502 (0.640, 9.780)	0.207 (0.044, 0.985)^*^	1.135 (0.824, 1.562)	2.89E-09 (3.21e-19, 26.05)	0.0947 (<0.001698, 12.85)	1.463 (0.389, 5.495)	2.508 (0.993, 6.335)
	(18.5, 25)	0.864 (0.732, 1.021)	0.966 (0.812, 1.149)	1.130 (0.997, 1.281)	0.709 (0.504, 0.997)^*^	0.943 (0.723, 1.229)	0.898 (0.750, 1.077)	0.966 (0.765, 1.220)
	(25, 30)	0.725 (0.555, 0.947)^*^	0.865 (0.748, 1.000)	0.881 (0.714, 1.087)	0.710 (0.500, 1.010)	0.811 (0.580, 1.133)	0.683 (0.528, 0.883)^**^	1.175 (1.006, 1.371)^*^
	>30	0.852 (0.760, 0.954)^**^	0.824 (0.712, 0.954)^*^	0.877 (0.750, 1.025)	0.644 (0.450, 0.923)^*^	0.668 (0.518, 0.862)^**^	0.732 (0.624, 0.858)^***^	1.339 (1.211, 1.481)^***^
Race/ethnicity	Non-Hispanic white	0.932 (0.831, 1.046)	0.897 (0.740, 1.086)	0.944 (0.704, 1.265)	0.889 (0.705, 1.120)	0.820 (0.538, 1.250)	0.878 (0.755, 1.022)	1.104 (0.865, 1.409)
	Non-Hispanic black	1.007 (0.810, 1.252)	0.746 (0.569, 0.980)^*^	0.918 (0.746, 1.129)	0.894 (0.664, 1.202)	0.860 (0.626, 1.181)	0.835 (0.648, 1.075)	1.134 (0.913, 1.407)
	Mexican American	0.820 (0.720, 0.933)^**^	0.872 (0.760, 1.001)	0.966 (0.834, 1.120)	0.601 (0.423, 0.855)^**^	0.785 (0.627, 0.983)^*^	0.763 (0.650, 0.896)^**^	1.274 (1.140, 1.423)^***^
	Others Hispanic	0.785 (0.623, 0.989)^*^	0.981 (0.794, 1.212)	0.936 (0.786, 1.116)	0.818 (0.650, 1.029)	0.638 (0.444, 0.917)^*^	0.764 (0.587, 0.993)^*^	1.147 (1.015, 1.298)^*^
	Other race	0.774 (0.535, 1.120)	0.749 (0.472, 1.189)	1.274 (1.059, 1.532)^*^	0.412 (0.175, 0.970)^*^	0.827 (0.622, 1.101)	0.773 (0.515, 1.161)	1.127 (0.874, 1.454)
Education	Less than 9th grade	0.866 (0.696, 1.078)	0.798 (0.646, 0.986)^*^	0.911 (0.761, 1.091)	0.722 (0.264, 1.976)	0.461 (0.280, 0.758)^**^	0.755 (0.582, 0.978)^*^	1.352 (1.072, 1.705)^*^
	9-11th grade	0.846 (0.696, 1.030)	0.967 (0.783, 1.193)	0.984 (0.873, 1.109)	0.948 (0.790, 1.138)	0.899 (0.741, 1.091)	0.852 (0.693, 1.047)	1.134 (0.957, 1.342)
	High school graduate	0.760 (0.648, 0.892)^**^	0.763 (0.634, 0.918)^**^	0.929 (0.769, 1.121)	0.808 (0.584, 1.118)	0.845 (0.664, 1.075)	0.689 (0.564, 0.842)^***^	1.170 (1.031, 1.327)^*^
	Some college degree	0.896 (0.776, 1.034)	0.912 (0.771, 1.079)	1.005 (0.855, 1.183)	0.548 (0.409, 0.733)^***^	0.828 (0.626, 1.096)	0.832 (0.683, 1.013)	1.236 (1.084, 1.408)^**^
	College graduate or above	0.972 (0.729, 1.295)	0.921 (0.636, 1.334)	1.203 (0.967, 1.497)	0.571 (0.313, 1.040)^**^	0.420 (0.176, 1,000)^*^	0.769 (0.445, 1.331)	1.300 (1.018, 1.661)^*^
Cigarette smoking	No	0.765 (0.604, 0.969)^*^	0.885 (0.746, 1.050)	1.116 (1.005, 1.240)^*^	0.674 (0.500, 0.910)^*^	0.872 (0.716, 1.061)	0.791 (0.642, 0.974)^*^	1.153 (0.999, 1.330)^*^
	Yes	0.862 (0.786, 0.945)^**^	0.860 (0.765, 0.967)^*^	0.893 (0.790, 1.010)	0.694 (0.533, 0.8903)^**^	0.712 (0.566, 0.896)^**^	0.771 (0.684, 0.870)^***^	1.286 (1.170, 1.413)^***^
Alcohol drinking	No	0.614 (0.432, 0.872)^**^	0.844 (0.677, 1.053)	1.121 (0.971, 1.294)	0.519 (0.295, 0.914)^*^	0.879 (0.667, 1.158)	0.699 (0.537, 0.909)^**^	1.280 (1.084, 1.512)^**^
	Yes	0.872 (0.799, 0.951)^**^	0.876 (0.790, 0.972)^*^	0.947 (0.858, 1.048)	0.719 (0.580, 0.892)^**^	0.751 (0.619, 0.912)^**^	0.796 (0.710, 0.894)^***^	1.213 (1.116, 1.317)^***^
Marital status	Married	0.795 (0.682, 0.926)^**^	0.854 (0.693, 1.053)	0.936 (0.772, 1.136)	0.479 (0.311, 0.739)^**^	0.747 (0.550, 1.014)	0.720 (0.568, 0.913)^**^	1.208 (1.061, 1.376)^**^
	Widowed	0.757 (0.540, 1.061)	0.844 (0.539, 1.321)	0.821 (0.553, 1.220)	0.583 (0.207, 1.639)	0.805 (0.366, 1.774)	0.680 (0.395, 1.171)	1.517 (1.135, 2.026)^**^
	Divorced	0.845 (0.716, 0.997)^*^	0.835 (0.693, 1.005)	0.842 (0.666, 1.064)	0.484 (0.232, 1.012)	0.424 (0.251, 0.714)^**^	0.705 (0.555, 0.895)^**^	1.304 (1.095, 1.552)^**^
	Separated	0.802 (0.552, 1.166)	0.751 (0.473, 1.193)	1.061 (0.827, 1.361)	0.380 (0.155, 0.928)^*^	0.966 (0.443, 2.105)	0.683 (0.451, 1.035)	1.168 (0.894, 1.525)
	Never married	0.795 (0.637, 0.991)^*^	0.988 (0.838, 1.165)	1.106 (0.967, 1.264)	0.735 (0.562, 0.962)^*^	0.993 (0.852, 1.158)	0.857 (0.702, 1.046)	1.090 (0.916, 1.299)
	Living with partner	1.024 (0.828, 1.267)	0.846 (0.654, 1.093)	0.963 (0.776, 1.195)	1.023 (0.735, 1.424)	0.526 (0.266, 1.040)	0.909 (0.678, 1.219)	1.416 (1.103, 1.820)^**^
Income ratio	High income	0.706 (0.493, 1.010)	0.972 (0.809, 1.169)	1.105 (0.963, 1.267)	0.584 (0.409, 0.834)^**^	0.765 (0.519, 1.126)	0.774 (0.581, 1.030)	1.270 (1.079, 1.495)^**^
	Middle income	0.908 (0.813, 1.014)	0.837 (0.726, 0.965)^*^	1.009 (0.899, 1.132)	0.729 (0.523, 1.017)	0.781 (0.619, 0.985)^*^	0.830 (0.712, 0.966)^*^	1.193 (1.046, 1.360)^**^
	Low income	0.808 (0.695, 0.940)^*^	0.808 (0.690, 0.948)^**^	0.895 (0.753, 1.064)	0.736 (0.533, 1.018)	0.793 (0.637, 0.986)^*^	0.707 (0.593, 0.842)^***^	1.248 (1.099, 1.418)^***^
Diabetes	No	NA	NA	NA	NA	NA	NA	NA
	Yes	0.845 (0.774, 0.922)^***^	0.870 (0.789, 0.961)^**^	0.985 (0.900, 1.077)	0.686 (0.561, 0.839)^***^	0.785 (0.668, 0.923)^**^	0.781 (0.698, 0.874)^***^	1.232 (1.138, 1.334)^***^

NHANES, National Health and Nutrition Examination Survey; BMI, body mass index; MET, metabolic equivalent of task; VWPA, vigorous work physical activity; MWPA, moderate work physical activity; TPA, walking or bicycling for transportation; VRPA, vigorous recreational physical activity; MRPA, moderate recreational physical activity; TMET, total MET; NA, not available due to limited sample sizes. ^*^p < 0.05, ^**^p < 0.01, ^***^p < 0.001. Data are expressed as odds ratio (95% confidence interval).

By BMI categories, significance was noted in respondents with obesity, and all physical activity indices, except TPA, and sedentary behavior were consistently and significantly associated with depression in respondents with obesity. By race and ethnicity, Mexican Americans were more likely to benefit from increased physical activity and reduced sitting time relative to respondents of other races. The association of physical activity and sedentary behavior with depression was less likely confounded by education, smoking, and drinking.

### Interaction analyses

The interaction between covariates and physical activity and sedentary behavior in association with depression was summarized in Table 4. Except for alcohol drinking and race/ethnicity, the other covariates significantly and consistently interacted with different types or intensities of physical activity and sedentary behavior when predicting the risk for depression at a significance level of 1‰.

**Table 4:** Interaction between covariates on a categorical scale and physical activity and sedentary behavior in association with depression.

Subgroups	Interaction *p* value						
	VWPA	MWPA	TPA	VRPA	MRPA	TMET	Sitting Time
Age	<0.001^***^	<0.001^***^	<0.001^***^	<0.001^***^	<0.001^***^	<0.001^***^	<0.001^***^
Sex	<0.001^***^	<0.001^***^	<0.001^***^	<0.001^***^	<0.001^***^	<0.001^***^	<0.001^***^
BMI	<0.001^***^	<0.001^***^	<0.001^***^	<0.001^***^	<0.001^***^	<0.001^***^	<0.001^***^
Race/ethnicity	0.017^*^	0.014^*^	0.014^*^	0.017^*^	0.015^*^	0.017^*^	0.063^*^
Education	<0.001^***^	<0.001^***^	<0.001^***^	<0.001^***^	<0.001^***^	<0.001^***^	<0.001^***^
Cigarette smoking	<0.001^***^	<0.001^***^	<0.001^***^	<0.001^***^	<0.001^***^	<0.001^***^	<0.001^***^
Alcohol drinking	0.190	0.199	0.210	0.143	0.178	0.172	0.253
Marital status	<0.001^***^	<0.001^***^	<0.001^***^	<0.001^***^	<0.001^***^	<0.001^***^	<0.001^***^
Income ratio	<0.001^***^	<0.001^***^	<0.001^***^	<0.001^***^	<0.001^***^	<0.001^***^	<0.001^***^

NHANES, National Health and Nutrition Examination Survey; BMI, body mass index; MET, metabolic equivalent of task; VWPA, vigorous work physical activity; MWPA, moderate work physical activity; TPA, walking or bicycling for transportation; VRPA, vigorous recreational physical activity; MRPA, moderate recreational physical activity; TMET, total MET. *p < 0.05, **p < 0.01, ***p < 0.001. Diabetes was not included due to the limited number of respondents who were free of diagnosed diabetes.

### Mediation analyses

**Figure 2:**
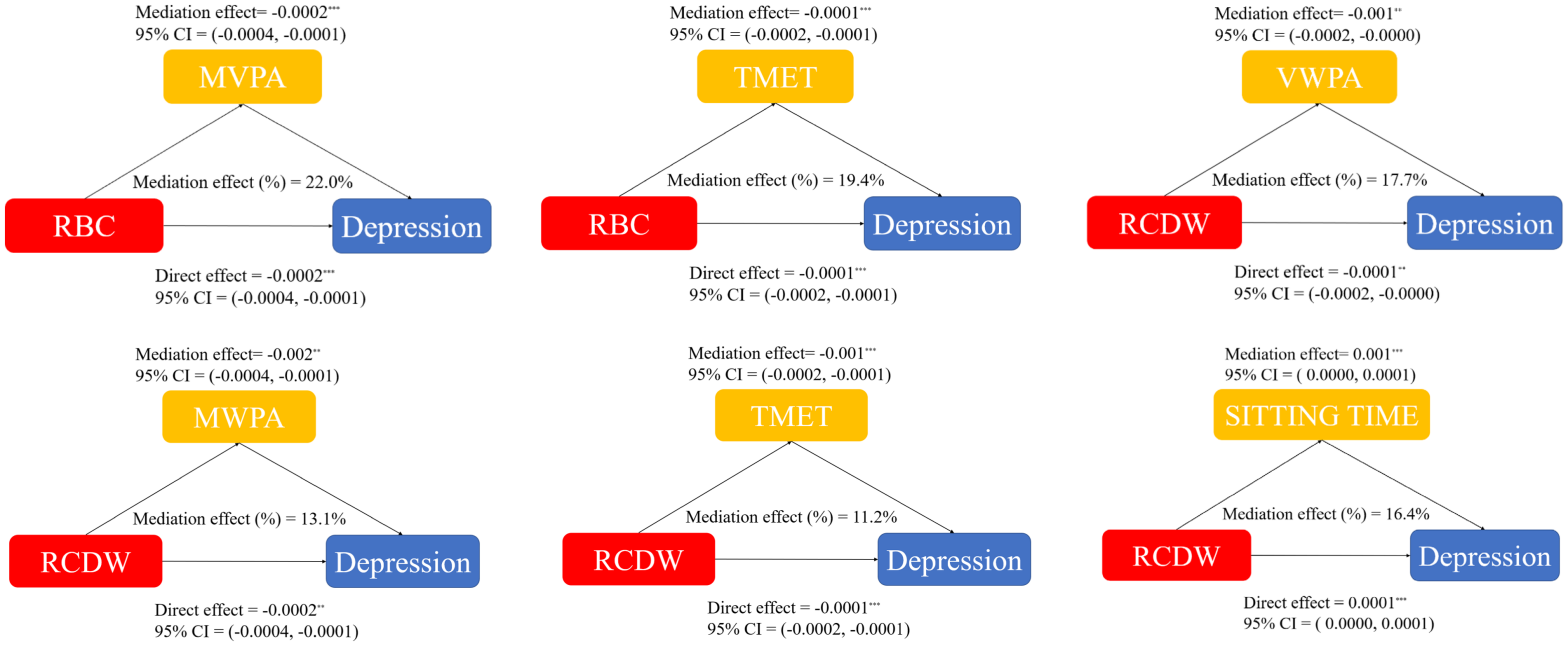
Mediation effect of red blood cell (RBC) and red cell distribution wide (RCDW) on the association of physical activity and sedentary behavior with depression. VWPA, vigorous work physical activity; MWPA, moderate work physical activity; TPA, walking or bicycling for transportation; VRPA, vigorous recreational physical activity; MRPA, moderate recreational physical activity; TMET, total metabolic equivalent of task; 95% CI, 95% confidence interval.

Figure 2 illustrates the mediation estimates of CBC that reached statistical significance on the association of physical activity and sedentary behavior with depression, and non-significant estimates were provided in Supplementary Materials (Supplementary Tables S1–S6).

Significance was observed for red blood cell (RBC) on TMET (19.4%) and MWPA (22.0%), and for red cell distribution wide (RCDW) on VWPA (17.7%), MWPA (13.1%), TMET (11.2%), and sitting time (16.4%) (*p* < 0.01 for all tests).

### Sensitivity and subgroup analyses

Supplementary Table S7 presented the overall association of physical activity and sedentary behavior with depression, additionally adjusting for antidepressant medication. After full adjustment, significance was merely noted for VRPA, MRPA, and sitting time. Grouping by the intake of antidepressant drugs, the association with depression was statistically significant for VRPA and MRPA in respondents not using antidepressant medication, and for MRPA in respondents using antidepressant medication (Supplementary Table S8).

## Discussion

In this study, we aimed to examine whether physical activity and sedentary behavior were associated with depression among 31,204 US adults from the NHANES 2007–2018, as well as the possible mediation impact of CBC on this association. Our findings indicate that more physical activity, except walking or bicycling for transportation, was significantly and independently associated with a lower risk of depression, and contrastingly, more sitting time can remarkably precipitate the occurrence of depression among adults. Moreover, our findings supported the mediatory role of RBC and RCDW in physical activity and sedentary behavior associated with depression. To the best of our knowledge, this is thus far the largest national study that has evaluated physical activity and sedentary behavior in predisposition to depression in adults.

Our findings that physical activity and sedentary behavior were significantly associated with depression are concordant with that of most prior studies (29, 30). As an extension, we considered different types or intensities of physical activity and observed heterogeneous associations with depression. For instance, we observed that moderate work physical activity was more beneficial in lowering the risk of depression than vigorous work physical activity, while moderate recreational physical activity was more beneficial for depression than vigorous recreational physical activity. This heterogeneity in magnitude is biologically plausible. It is widely recognized that the oxidative stress pathway is involved in the pathophysiological processes of depression (21), and this pathway can stimulate the production of pro-inflammatory cytokines and cause damage to neuroplasticity. The fact that physical activity can exert both positive and negative effects on inflammatory and redox status (31) can explain why different intensities of physical activity exert different effects on depression. Moreover, strenuous physical activity can increase the formation of reactive oxygen species and induce an acute phase immune response like infection, including cytokine release, activation of immune-reactive cell lines, and neutrophil initiation of the acute temporal phase response (32). Accordingly, the production and release of interleukin-1β, interleukin-6, and tumor necrosis factor-α were enhanced, and these biomarkers evolved into chronic inflammation and produced systemic inflammation (33). Furthermore, the increase of immune cell counts can elevate glucocorticoid levels, which can remodel the actin cytoskeleton of blood cells, impact cell morpho-rheological properties, and alter peripheral blood cell function (10).

In addition to the proposed biological explanations, there is evidence that the anti-depressive impact of physical activity relies on psychological and sociological factors (30). Generally, recreational physical activity can make people feel well because of autonomous decisions, which might explain why recreational physical activity differs from transport-related physical activity in predicting depression (34). Individuals with moderate levels of work engagement are usually well educated and have substantial income, and thus may be prone to depression. Thus, more physical activity may not always be beneficial, and the types, durations, and frequencies of exercise need to be considered (16). As such, we recommend the simultaneous consideration of both types and intensities of physical activity when assessing its association with mental health. Our results regarding various aspects of physical activity provide an evidence basis for clinicians in the assessment and treatment of patients with mental health disorders, particularly depression.

Another important finding of this study that merits discussion is that RBC and RCDW can significantly mediate the association between physical activity and depression. In fact, RBC and RCDW can reflect cell shape and function. RBC is a sensitive cell exposed to circulating inflammatory mediators and associated oxidative stresses (35). RCDW is a quantitative indicator of RBC size variation and has recently been established as a promising marker (36). Especially in the pathogenesis of depression, high RCDW can harbinger the inflammatory state, which was found to be associated with high depression scores (37). Another study reported a decrease in RBC in depressive patients compared to normal controls; however, RCDW was comparable between the two groups (38). In this study, we found that RCDW can mediate the association of physical activity and sedentary behavior, especially TMET, with depression. Prior studies have examined the correlation between RCDW and sedentary behavior, physical activity (39), and depression (40), but a literature search has failed to reveal any supportive evidence on the mediatory role of RCDW in the association between exercise or sitting and depression. We agree that more well-designed, longitudinal studies are required to confirm our findings.

Additionally, in this study, sedentary behavior was also found to be associated with depression risk, consistent with the results of prior studies (31). Evidence from the NHANES 2007–2012 database revealed that television viewing and computer use were found to be two major forms of sedentary behavior (41), which can indirectly support the close association between sitting time and depression observed in this study. In support of this note, decreasing screen time was found to be effective to some extent in improving depression (42). Hence, special attention should be paid to subjects at high risk for depression and with poor sedentary behavior, including pregnant women and older adults (43).

Finally, several limitations should be acknowledged. First, this study is cross-sectional in nature, and the cause-and-effect between physical activity or sedentary behavior and depression cannot be addressed. Further meta-analyses or bibliometric analyses are expected to consolidate the observations of this study. Second, depression and physical activity were self-reported, leaving the possibility of recall bias an open question. The PHQ-9 adopted in this study has been widely used to define depression in clinical and epidemiological settings due to its high sensitivity and specificity (44, 45). Third, only US adults were analyzed, and extrapolation of our findings to other racial or ethnic groups should be made with caution. We agree that future longitudinal studies focusing on the relationship between physical activity, sedentary behavior, and CBC parameters with depression could validate the outcomes of this study.

In conclusion, our findings indicate that more physical activity and less sitting time were associated with a lower likelihood of having depression among US adults, and this association was probably mediated by RBC and RCDW. Further functional studies are warranted to elucidate molecular mechanisms for the involvement of physical activity and sedentary behavior in the pathogenesis of mental health.

## Data availability statement

The original contributions presented in the study are included in the article/Supplementary material, further inquiries can be directed to the corresponding authors.

## Author contributions

YM: Writing – review & editing, Writing – original draft. NM: Writing – original draft. YS: Writing – original draft. NZ: Writing – original draft. JW: Writing – review & editing. XC: Writing – review & editing. WN: Writing – review & editing.

## Footnotes

^1^ https://vizhub.healthdata.org/gbd-results/

^2^ https://wwwn.cdc.gov/Nchs/Nhanes/2007-2008/PAQ_E.htm
