# Supplementary material for: The association of physical activity and sedentary behavior with depression in US adults: NHANES 2007–2018
Source: Front Public Health. 2024 Jun 21;12:1404407. doi: 10.3389/fpubh.2024.1404407 (PMC11224452; doi:10.3389/fpubh.2024.1404407)
Supplement: Supplementary file 1 [file Data_Sheet_1.docx]

**Table S1.** VWPA associated with depression

| **Variables** | **Total effect** | | | |  | **Natural direct effect** | | | |  | **Natural indirect effect** | | | | **Proportion** | ***P*** |
| --- | --- | --- | --- | --- | --- | --- | --- | --- | --- | --- | --- | --- | --- | --- | --- | --- |
|  | **β** | **Lower** | **Upper** | ***P*** |  | **β** | **Lower** | **Upper** | ***P*** |  | **β** | **Lower** | **Upper** | ***P*** |  |  |
| **WBC** | -.0001 | -.0002 | -.0000 | 0.003 |  | -.0001 | -.0002 | -.0000 | 0.003 |  | 8.87e-07 | -1.12e-06 | 2.90e-06 | 0.387 | -5.7% | 0.032 |
| **LYMper** | -.0001 | -.0002 | -.0001 | <0.001 |  | -.0001 | -.0002 | -.0001 | <0.001 |  | -4.72e-07 | -1.78e-06 | 8.40e-07 | 0.481 | 1.2% | 0.323 |
| **MONper** | -.0001 | -.0002 | -.0001 | <0.001 |  | -.0001 | -.0002 | -.0001 | <0.001 |  | 4.71e-07 | -1.35e-06 | 2.29e-06 | 0.612 | 6.3% | 0.018 |
| **segNEUper** | -.0001 | -.0002 | -.0001 | <0.001 |  | -.0001 | -.0002 | -.0001 | <0.001 |  | -9.98e-08 | -1.44e-06 | 1.24e-06 | 0.884 | 3.7% | 0.055 |
| **EOSper** | -.0001 | -.0002 | -.0001 | <0.001 |  | -.0001 | -.0002 | -.0001 | <0.001 |  | 2.11e-08 | -7.28e-07 | 7.70e-07 | 0.956 | 1.1% | 0.235 |
| **BASper** | -.0001 | -.0002 | -.0001 | <0.001 |  | -.0001 | -.0002 | -.0001 | <0.001 |  | 8.61e-08 | -8.68e-07 | 1.04e-06 | 0.860 | -0.0% | 0.924 |
| **LYMnum** | -.0001 | -.0002 | -.0001 | <0.001 |  | -.0001 | -.0002 | -.0001 | <0.001 |  | -2.06e-07 | -8.57e-07 | 4.46e-07 | 0.536 | -0.4% | 0.568 |
| **MONnum** | -.0001 | -.0002 | -.0001 | <0.001 |  | -.0001 | -.0002 | -.0001 | <0.001 |  | -1.12e-08 | -6.57e-07 | 6.35e-07 | 0.973 | -4.1% | 0.022 |
| **NEUnum** | -.0001 | -.0002 | -.0001 | <0.001 |  | -.0001 | -.0002 | -.0001 | <0.001 |  | 1.34e-06 | -9.71e-07 | 3.66e-06 | 0.256 | -9.5% | 0.016 |
| **EOSnum** | -.0001 | -.0002 | -.0001 | <0.001 |  | -.0001 | -.0002 | -.0001 | <0.001 |  | 6.61e-08 | -6.11e-07 | 7.43e-07 | 0.848 | -1.8% | 0.117 |
| **BASnum** | -.0001 | -.0002 | -.0001 | <0.001 |  | -.0001 | -.0002 | -.0001 | <0.001 |  | 2.36e-07 | -4.75e-07 | 9.47e-07 | 0.516 | -3.2% | 0.074 |
| **RBC** | -.0001 | -.0002 | -.0001 | <0.001 |  | -.0001 | -.0002 | -.0001 | <0.001 |  | -6.04e-07 | -2.00e-06 | 7.90e-07 | 0.396 | 43.2% | <0.001 |
| **Hemoglobin** | -.0071 | .0018 | -.0108 | -.0035 |  | -.0070 | -.0106 | -.0034 | <0.001 |  | -.0001 | -.0003 | 2.26e-06 | 0.054 | 124.9% | <0.001 |
| **Hematocrit** | -.0002 | -.0003 | -.0001 | 0.001 |  | -.0002 | -.0003 | -.0001 | 0.001 |  | 3.12e-08 | -1.50e-06 | 1.56e-06 | 0.968 | 31.8% | <0.001 |
| **MCV** | -.0001 | -.0002 | -.0001 | <0.001 |  | -.0001 | -.0002 | -.0001 | <0.001 |  | 5.28e-07 | -1.41e-06 | 2.46e-06 | 0.593 | 0.5% | 0.594 |
| **MCH** | -.0071 | -.0115 | -.0028 | 0.001 |  | -.0072 | -.0115 | -.0028 | 0.001 |  | .0000 | -.0001 | .0001 | 0.443 | 6% | 0.034 |
| **MCHC** | -.0001 | -.0002 | -.0001 | <0.001 |  | -.0001 | -.0002 | -.0001 | <0.001 |  | -1.49e-06 | -3.55e-06 | 5.58e-07 | 0.154 | 4.9% | 0.029 |
| **RCDW** | -.0001 | -.0002 | -.0000 | 0.002 |  | -.0001 | -.0002 | -.0000 | 0.002 |  | -3.58e-06 | -6.64e-06 | -5.17e-07 | 0.022 | 17.7% | <0.001 |
| **PLT** | -.0001 | -.0002 | -.0001 | <0.001 |  | -.0001 | -.0002 | -.0001 | <0.001 |  | 1.29e-07 | -7.52e-07 | 1.01e-06 | 0.774 | 3.8% | 0.152 |
| **MPV** | -.0001 | -.0002 | -.0001 | <0.001 |  | -.0001 | -.0002 | -.0001 | <0.001 |  | 1.31e-07 | -8.54e-07 | 1.12e-06 | 0.795 | 0.5% | 0.425 |

**Table S2.** MWPA associated with depression

| **Variables** | **Total effect** | | | | **Natural direct effect** | | | | **Natural indirect effect** | | | | **Proportion** | ***P*** |
| --- | --- | --- | --- | --- | --- | --- | --- | --- | --- | --- | --- | --- | --- | --- |
|  | **β** | **Lower** | **Upper** | ***P*** | **β** | **Lower** | **Upper** | ***P*** | **β** | **Lower** | **Upper** | ***P*** |  |  |
| **WBC** | -.0002 | -.0004 | -.0001 | <0.001 | -.0002 | -.0004 | -.0001 | <0.001 | -3.29e-07 | -2.01e-06 | 1.35e-06 | 0.701 | -2.3% | 0.264 |
| **LYMper** | -.0002 | -.0003 | -.0002 | <0.001 | -.0002 | -.0003 | -.0001 | <0.001 | -2.28e-06 | -6.13e-06 | 1.57e-06 | 0.245 | 2.0% | 0.073 |
| **MONper** | -.0002 | -.0004 | -.0001 | <0.001 | -.0002 | -.0004 | -.0001 | <0.001 | 9.50e-08 | -1.06e-06 | 1.25e-06 | 0.16 | 2.0% | 0.326 |
| **segNEUper** | -.0002 | -.0004 | -.0001 | <0.001 | -.0002 | -.0004 | -.0001 | <0.001 | -1.79e-06 | -4.36e-06 | 7.82e-07 | 0.172 | 3.0% | 0.048 |
| **EOSper** | -.0002 | -.0004 | -.0001 | <0.001 | -.0002 | -.0004 | -.0001 | <0.001 | 6.68e-08 | -1.59e-06 | 1.72e-06 | 0.937 | 0.1% | 0.833 |
| **BASper** | -.0002 | -.0004 | -.0001 | <0.001 | -.0002 | -.0004 | -.0001 | <0.001 | -5.69e-09 | -1.46e-06 | 1.45e-06 | 0.994 | 0.0% | 0.974 |
| **LYMnum** | -.0002 | -.0004 | -.0002 | <0.001 | -.0002 | -.0004 | -.0001 | <0.001 | -3.75e-07 | -1.54e-06 | 7.91e-07 | 0.528 | -0.3% | 0.522 |
| **MONnum** | -.0002 | -.0004 | -.0001 | <0.001 | -.0002 | -.0004 | -.0001 | <0.001 | -2.27e-08 | -1.15e-06 | 1.11e-06 | 0.969 | -1.8% | 0.062 |
| **NEUnum** | -.0002 | -.0004 | -.0001 | <0.001 | -.0002 | -.0004 | -.0001 | <0.001 | -1.32e-06 | -4.17e-06 | 1.52e-06 | 0.361 | -2.2% | 0.463 |
| **EOSnum** | -.0002 | -.0003 | -.0001 | <0.001 | -.0002 | -.0003 | -.0001 | <0.001 | -6.04e-08 | -1.25e-06 | 1.13e-06 | 0.921 | -0.4% | 0.485 |
| **BASnum** | -.0002 | -.0004 | -.0001 | <0.001 | -.0002 | -.0004 | -.0001 | <0.001 | 2.91e-07 | -1.02e-06 | 1.60e-06 | 0.664 | -2.2% | 0.107 |
| **RBC** | -.0002 | -.0004 | -.0001 | <0.001 | -.0002 | -.0004 | -.0001 | <0.001 | -2.70e-06 | -5.99e-06 | 6.03e-07 | 0.109 | 22.0% | <0.001 |
| **Hemoglobin** | -.0002 | -.0004 | -.0000 | <0.001 | -.0002 | -.0004 | -.0001 | <0.001 | -9.88e-07 | -4.16e-06 | 2.18e-06 | 0.541 | 28.0% | <0.001 |
| **Hematocrit** | -.0095 | -.0144 | -.0045 | 0.017 | -.0002 | -.0004 | -.0000 | 0.017 | 1.09e-07 | -3.68e-06 | 3.90e-06 | 0.955 | 20.3% | <0.001 |
| **MCV** | -.0002 | -.0004 | -.0001 | <0.001 | -.0002 | -.0004 | -.0001 | <0.001 | -8.82e-07 | -4.00e-06 | 2.23e-06 | 0.579 | 0.0% | 0.951 |
| **MCH** | -.0002 | -.0004 | -.0001 | <0.001 | -.0002 | -.0004 | -.0001 | <0.001 | -1.74e-08 | -1.55e-06 | 1.52e-06 | 0.982 | 0.7% | 0.296 |
| **MCHC** | -.0003 | -.0004 | -.0001 | <0.001 | -.0002 | -.0004 | -.0001 | <0.001 | -1.27e-06 | -3.91e-06 | 1.37e-06 | 0.347 | 3.2% | 0.034 |
| **RCDW** | -.0002 | -.0004 | -.0001 | 0.001 | -.0002 | -.0004 | -.0001 | 0.001 | -3.41e-06 | -7.92e-06 | 1.10e-06 | 0.138 | 13.1% | <0.001 |
| **PLT** | -.0002 | -.0004 | -.0001 | <0.001 | -.0002 | -.0004 | -.0001 | <0.001 | 4.31e-07 | -1.44e-06 | 2.30e-06 | 0.651 | -3.2% | 0.135 |
| **MPV** | -.0002 | -.0004 | -.0001 | <0.001 | -.0002 | -.0004 | -.0001 | <0.001 | 2.18e-07 | -2.16e-06 | 2.60e-06 | 0.857 | 0.4% | 0.439 |

**Table S3.** VRPA associated with depression

| **Variables** | **Total effect** | | | | **Natural direct effect** | | | | **Natural indirect effect** | | | | **Proportion** | ***P*** |
| --- | --- | --- | --- | --- | --- | --- | --- | --- | --- | --- | --- | --- | --- | --- |
|  | **β** | **Lower** | **Upper** | ***P*** | **β** | **Lower** | **Upper** | ***P*** | **β** | **Lower** | **Upper** | ***P*** |  |  |
| **WBC** | -.0012 | -.0017 | -.0008 | <0.001 | -.0012 | -.0017 | -.0008 | <0.001 | -5.32e-06 | -.0000 | 4.49e-06 | 0.288 | 3.4% | <0.001 |
| **LYMper** | -.0012 | -.0017 | -.0007 | <0.001 | -.0012 | -.0017 | -.0007 | <0.001 | -.0000 | -.0000 | 2.66e-06 | 0.098 | 1.9% | 0.004 |
| **MONper** | -.0012 | -.0017 | -.0008 | <0.001 | -.0012 | -.0017 | -.0008 | <0.001 | 5.88e-08 | -6.09e-06 | 6.21e-06 | 0.985 | 2.4% | <0.001 |
| **segNEUper** | -.0012 | -.0017 | -.0008 | <0.001 | -.0012 | -.0016 | -.0008 | <0.001 | -8.78e-06 | -.0000 | 4.88e-06 | 0.208 | 3.1% | <0.001 |
| **EOSper** | -.0012 | -.0016 | -.0008 | <0.001 | -.0012 | -.0016 | -.0008 | <0.001 | -3.02e-06 | -.0000 | 5.53e-06 | 0.489 | -0.1% | 0.361 |
| **BASper** | -.0012 | -.0016 | -.0009 | <0.001 | -.0012 | -.0016 | -.0009 | <0.001 | -1.07e-06 | -.0000 | .0000 | 0.890 | -0.0% | 0.959 |
| **LYMnum** | -.0012 | -.0017 | -.0008 | <0.001 | -.0012 | -.0017 | -.0008 | <0.001 | -8.58e-07 | -5.60e-06 | 3.88e-06 | 0.723 | 0.1% | 0.302 |
| **MONnum** | -.0012 | -.0017 | -.0008 | <0.001 | -.0012 | -.0017 | -.0008 | <0.001 | -1.27e-07 | -9.60e-06 | 9.34e-06 | 0.979 | 0.7% | 0.033 |
| **NEUnum** | -.0012 | -.0017 | -.0008 | <0.001 | -.0012 | -.0017 | -.0008 | <0.001 | -9.78e-06 | -.0000 | 6.55e-06 | 0.240 | 7.3% | <0.001 |
| **EOSnum** | -.0012 | -.0016 | -.0008 | <0.001 | -.0012 | -.0016 | -.0008 | <0.001 | -9.57e-07 | -.0000 | .0000 | 0.865 | 0.5% | 0.136 |
| **BASnum** | -.0012 | -.0016 | -.0008 | <0.001 | -.0012 | -.0016 | -.0008 | <0.001 | -4.21e-06 | -.0000 | 6.16e-06 | 0.426 | 1.8% | <0.001 |
| **RBC** | -.0012 | -.0017 | -.0008 | <0.001 | -.0012 | -.0017 | -.0008 | <0.001 | -9.67e-06 | -.0000 | 3.91e-06 | 0.163 | 6.2% | <0.001 |
| **Hemoglobin** | -.0012 | -.0016 | -.0008 | <0.001 | -.0012 | -.0016 | -.0008 | <0.001 | -3.64e-06 | -.0000 | .0000 | 0.640 | 6.5% | <0.001 |
| **Hematocrit** | -.0010 | -.0015 | -.0005 | <0.001 | -.0010 | -.0015 | -.0005 | <0.001 | 9.28e-07 | -.0000 | .0000 | 0.930 | 9.2% | <0.001 |
| **MCV** | -.0002 | -.0004 | -.0001 | <0.001 | -.0002 | -.0004 | -.0001 | <0.001 | -8.82e-07 | -4.00e-06 | 2.23e-06 | 0.579 | 0.0% | 0.951 |
| **MCH** | -.0012 | -.0016 | -.0008 | <0.001 | -.0012 | -.0016 | -.0008 | <0.001 | -2.45e-08 | -8.01e-06 | 7.96e-06 | 0.995 | -0.1% | 0.299 |
| **MCHC** | -.0012 | -.0018 | -.0007 | <0.001 | -.0012 | -.0018 | -.0007 | <0.001 | -1.00e-07 | -9.55e-06 | 9.35e-06 | 0.983 | 0.3% | 0.141 |
| **RCDW** | -.0012 | -.0017 | -.0007 | <0.001 | -.0012 | -.0017 | -.0007 | <0.001 | -.0000 | -.0001 | -.0000 | 0.004 | 6.3% | <0.001 |
| **PLT** | -.0012 | -.0016 | -.0009 | <0.001 | -.0012 | -.0016 | -.0009 | <0.001 | -1.44e-06 | -.0000 | .0000 | 0.809 | 2.8% | <0.001 |
| **MPV** | -.0012 | -.0019 | -.0008 | <0.001 | -.0012 | -.0017 | -.0008 | <0.001 | -3.01e-07 | -6.49e-06 | 5.89e-06 | 0.924 | -0.1% | 0.391 |

**Table S4.** MRPA associated with depression

| **Variables** | **Total effect** | | | | **Natural direct effect** | | | | **Natural indirect effect** | | | | **Proportion** | ***P*** |
| --- | --- | --- | --- | --- | --- | --- | --- | --- | --- | --- | --- | --- | --- | --- |
|  | **β** | **Lower** | **Upper** | ***P*** | **β** | **Lower** | **Upper** | ***P*** | **β** | **Lower** | **Upper** | ***P*** |  |  |
| **WBC** | -.0012 | -.0015 | -.0008 | <0.001 | -.0012 | -.0015 | -.0008 | <0.001 | -4.54e-06 | -.0000 | 4.06e-06 | 0.301 | 2.4% | <0.001 |
| **LYMper** | -.0012 | -.0015 | -.0008 | <0.001 | -.0012 | -.0015 | -.0008 | <0.001 | -9.43e-06 | -.0000 | 4.78e-06 | 0.193 | 0.7% | 0.042 |
| **MONper** | -.0012 | -.0016 | -.0008 | <0.001 | -.0012 | -.0016 | -.0008 | <0.001 | 2.91e-07 | -5.08e-06 | 5.66e-06 | 0.915 | 1.9% | 0.003 |
| **segNEUper** | -.0012 | -.0015 | -.0008 | <0.001 | -.0017 | -.0015 | -.0008 | <0.001 | -6.20e-06 | -.0000 | 4.76e-06 | 0.268 | 1.2% | 0.010 |
| **EOSper** | -.0012 | -.0015 | -.0008 | <0.001 | -.0012 | -.0015 | -.0008 | <0.001 | -1.71e-06 | -7.18e-06 | 3.75e-06 | 0.539 | -0.0% | 0.703 |
| **BASper** | -.0012 | -.0015 | -.0009 | <0.001 | -.0012 | -.0015 | -.0009 | <0.001 | 8.75e-08 | -4.59e-06 | 4.77e-06 | 0.971 | -0.0% | 0.929 |
| **LYMnum** | -.0011 | -.0015 | -.0008 | <0.001 | -.0012 | -.0015 | -.0008 | <0.001 | -1.28e-06 | -7.60e-06 | 5.04e-06 | 0.692 | 0.2% | 0.337 |
| **MONnum** | -.0012 | -.0016 | -.0007 | <0.001 | -.0012 | -.0016 | -.0007 | <0.001 | -1.81e-08 | -7.77e-06 | 7.73e-06 | 0.996 | 0.5% | 0.084 |
| **NEUnum** | -.0012 | -.0016 | -.0008 | <0.001 | -.0012 | -.0016 | -.0008 | <0.001 | -5.60e-07 | -7.81e-06 | 6.69e-06 | 0.880 | 0.4% | 0.137 |
| **EOSnum** | -.0007 | -.0010 | -.0004 | <0.001 | -.0007 | -.0010 | -.0004 | <0.001 | -1.55e-06 | -5.42e-06 | 2.32e-06 | 0.433 | 0.6% | 0.049 |
| **BASnum** | -.0012 | -.0016 | -.0007 | <0.001 | -.0012 | -.0016 | -.0007 | <0.001 | -8.11e-07 | -5.87e-06 | 4.25e-06 | 0.754 | 0.8% | 0.061 |
| **RBC** | -.0012 | -.0016 | -.0008 | <0.001 | -.0012 | -.0016 | -.0008 | <0.001 | -2.09e-07 | -7.93e-06 | 7.51e-06 | 0.958 | 2.2% | <0.001 |
| **Hemoglobin** | -.0012 | -.0016 | -.0008 | <0.001 | -.0012 | -.0016 | -.0008 | <0.001 | -1.26e-06 | -5.21e-06 | 2.70e-06 | 0.533 | 4.4% | <0.001 |
| **Hematocrit** | -.0011 | -.0017 | -.0004 | 0.001 | -.0011 | -.0017 | -.0004 | 0.001 | 5.08e-08 | -5.21e-06 | 5.31e-06 | 0.985 | 5.3% | <0.001 |
| **MCV** | -.0012 | -.0015 | -.0009 | <0.001 | -.0012 | -.0015 | -.0009 | <0.001 | 3.59e-06 | -4.69e-06 | .0000 | 0.395 | 0.1% | 0.680 |
| **MCH** | -.0012 | -.0015 | -.0008 | <0.001 | -.0012 | -.0015 | -.0008 | <0.001 | 2.49e-06 | -4.24e-06 | 9.22e-06 | 0.468 | 0.4% | 0.313 |
| **MCHC** | -.0012 | -.0016 | -.0008 | <0.001 | -.0012 | -.0016 | -.0008 | <0.001 | -1.97e-06 | -9.21e-06 | 5.27e-06 | 0.594 | 0.6% | 0.062 |
| **RCDW** | -.0012 | -.0015 | -.0009 | <0.001 | -.0012 | -.0015 | -.0009 | <0.001 | -5.63e-06 | -.0000 | 7.08e-06 | 0.385 | 3.5% | <0.001 |
| **PLT** | -.0012 | -.0015 | -.0008 | <0.001 | -.0012 | -.0015 | -.0008 | <0.001 | -3.91e-07 | -5.64e-06 | 4.86e-06 | 0.884 | 2.8% | <0.001 |
| **MPV** | -.0012 | -.0015 | -.0008 | <0.001 | -.0012 | -.0015 | -.0008 | <0.001 | -1.37e-07 | -3.80e-06 | 3.53e-06 | 0.942 | -0.0% | 0.675 |

**Table S5.** TMET associated with depression

| **Variables** | **Total effect** | | | | **Natural direct effect** | | | | **Natural indirect effect** | | | | **Proportion** | ***P*** |
| --- | --- | --- | --- | --- | --- | --- | --- | --- | --- | --- | --- | --- | --- | --- |
|  | **β** | **Lower** | **Upper** | ***P*** | **β** | **Lower** | **Upper** | ***P*** | **β** | **Lower** | **Upper** | ***P*** |  |  |
| **WBC** | -.0001 | -.0002 | -.0001 | <0.001 | -.0001 | -.0002 | -.0001 | <0.001 | 5.78e-08 | -5.79e-07 | 6.95e-07 | 0.859 | -0.3% | 0.809 |
| **LYMper** | -.0001 | -.0002 | -.0001 | <0.001 | -.0001 | -.0002 | -.0001 | <0.001 | -1.10e-06 | -2.78e-06 | 5.84e-07 | 0.201 | 1.8% | 0.010 |
| **MONper** | -.0001 | -.0002 | -.0001 | <0.001 | -.0001 | -.0002 | -.0001 | <0.001 | 2.36e-07 | -4.17e-07 | 8.88e-07 | 0.479 | 3.3% | 0.003 |
| **segNEUper** | -.0001 | -.0002 | -.0001 | <0.001 | -.0001 | -.0002 | -.0001 | <0.001 | -6.92e-07 | -1.98e-06 | 5.97e-07 | 0.293 | 3.3% | 0.001 |
| **EOSper** | -.0001 | -.0002 | -.0001 | <0.001 | -.0001 | -.0002 | -.0001 | <0.001 | -7.24e-08 | -7.53e-07 | 6.08e-07 | 0.835 | 0.3% | 0.279 |
| **BASper** | -.0001 | -.0002 | -.0001 | <0.001 | -.0001 | -.0002 | -.0001 | <0.001 | 3.14e-08 | -4.86e-07 | 5.49e-07 | 0.905 | -0.0% | 0.881 |
| **LYMnum** | -.0001 | -.0002 | -.0001 | <0.001 | -.0001 | -.0002 | -.0001 | <0.001 | -1.74e-07 | -7.88e-07 | 4.40e-07 | 0.579 | -0.1% | 0.695 |
| **MONnum** | -.0001 | -.0002 | -.0001 | <0.001 | -.0001 | -.0002 | -.0001 | <0.001 | -1.26e-08 | -7.50e-07 | 7.25e-07 | 0.973 | -1.0% | 0.051 |
| **NEUnum** | -.0001 | -.0002 | -.0001 | <0.001 | -.0001 | -.0002 | -.0001 | <0.001 | -1.34e-07 | -1.47e-06 | 1.20e-06 | 0.844 | 0.9% | 0.563 |
| **EOSnum** | -.0001 | -.0002 | -.0001 | <0.001 | -.0001 | -.0002 | -.0001 | <0.001 | -3.20e-08 | -4.31e-07 | 3.67e-07 | 0.875 | -0.3% | 0.296 |
| **BASnum** | -.0001 | -.0002 | -.0001 | <0.001 | -.0001 | -.0002 | -.0001 | <0.001 | 1.28e-07 | -4.62e-07 | 7.18e-07 | 0.671 | -0.5% | 0.453 |
| **RBC** | -.0001 | -.0002 | -.0001 | <0.001 | -.0001 | -.0002 | -.0001 | <0.001 | -9.42e-07 | -2.14e-06 | 2.59e-07 | 0.124 | 19.4% | <0.001 |
| **Hemoglobin** | -.0001 | -.0002 | -.0001 | <0.001 | -.0001 | -.0002 | -.0001 | <0.001 | -4.54e-07 | -1.96e-06 | 1.05e-06 | 0.555 | 33.7% | <0.001 |
| **Hematocrit** | -.0002 | -.0002 | -.0001 | <0.001 | -.0002 | -.0002 | -.0001 | <0.001 | 1.08e-07 | -1.89e-06 | 2.10e-06 | 0.915 | 20.5% | <0.001 |
| **MCV** | -.0001 | -.0002 | -.0001 | <0.001 | -.0001 | -.0002 | -.0001 | <0.001 | 2.72e-07 | -1.16e-06 | 1.71e-06 | 0.711 | 0.1% | 0.614 |
| **MCH** | -.0001 | -.0002 | -.00001 | <0.001 | -.0001 | -.0002 | -.0001 | <0.001 | 4.85e-07 | -7.86e-07 | 1.76e-06 | 0.454 | 0.7% | 0.282 |
| **MCHC** | -.0001 | -.0002 | -.0001 | <0.001 | -.0001 | -.0002 | -.0001 | <0.001 | -1.05e-06 | -2.78e-06 | 6.75e-07 | 0.232 | 2.4% | 0.036 |
| **RCDW** | -.0001 | -.0002 | -.0001 | <0.001 | -.0001 | -.0002 | -.0001 | <0.001 | -3.08e-06 | -5.93e-06 | -2.30e-07 | 0.034 | 11.2% | <0.001 |
| **PLT** | -.0001 | -.0002 | -.0001 | <0.001 | -.0001 | -.0002 | -.0001 | <0.001 | 1.14e-07 | -7.44e-07 | 9.72e-07 | 0.795 | 2.0% | 0.078 |
| **MPV** | -.0001 | -.0002 | -.0001 | <0.001 | -.0001 | -.0002 | -.0001 | <0.001 | 1.39e-07 | -1.10e-06 | 1.38e-06 | 0.826 | 0.2% | 0.447 |

**Table S6.** Sitting time associated with depression

| **Variables** | **Total effect** | | | | **Natural direct effect** | | | | **Natural indirect effect** | | | | **Proportion** | ***P*** |
| --- | --- | --- | --- | --- | --- | --- | --- | --- | --- | --- | --- | --- | --- | --- |
|  | **β** | **Lower** | **Upper** | ***P*** | **β** | **Lower** | **Upper** | ***P*** | **β** | **Lower** | **Upper** | ***P*** |  |  |
| **WBC** | .0001 | .0000 | .0001 | <0.001 | .0001 | .0000 | .0001 | <0.001 | 6.76e-07 | -2.53e-07 | 1.61e-06 | 0.154 | 3.3% | 0.005 |
| **LYMper** | .0001 | .0000 | .0001 | <0.001 | .0001 | .0000 | .0001 | <0.001 | 6.48e-07 | -1.23e-07 | 1.42e-06 | 0.099 | 3.5% | 0.002 |
| **MONper** | .0001 | .0000 | .0001 | <0.001 | .0001 | .0000 | .0001 | <0.001 | 4.40e-08 | -2.02e-07 | 2.90e-07 | 0.727 | -3.9% | 0.002 |
| **segNEUper** | .0001 | .0000 | .0001 | <0.001 | .0001 | .0000 | .0001 | <0.001 | 5.82e-07 | -3.82e-07 | 1.55e-06 | 0.237 | 4.2% | <0.001 |
| **EOSper** | .0001 | .0000 | .0001 | <0.001 | .0001 | .0000 | .0001 | <0.001 | -8.47e-08 | -4.50e-07 | 2.81e-07 | 0.650 | 0.2% | 0.386 |
| **BASper** | .0001 | .0000 | .0001 | <0.001 | .0001 | .0000 | .0001 | <0.001 | 6.23e-09 | -1.85e-07 | 1.97e-07 | 0.949 | 0.2% | 0.633 |
| **LYMnum** | .0001 | .0000 | .0001 | <0.001 | .0001 | .0000 | .0001 | <0.001 | -7.10e-08 | -4.05e-07 | 2.63e-07 | 0.677 | -0.4% | 0.280 |
| **MONnum** | .0001 | .0000 | .0001 | <0.001 | .0001 | .0000 | .0001 | <0.001 | -2.36e-08 | -6.38e-07 | 5.91e-07 | 0.940 | 2.7% | 0.020 |
| **NEUnum** | .0001 | .0000 | .0001 | <0.001 | .0001 | .0000 | .0001 | <0.001 | 1.24e-06 | -3.67e-07 | 2.85e-06 | 0.130 | 11.6% | <0.001 |
| **EOSnum** | .0001 | .0000 | .0001 | <0.001 | .0001 | .0000 | .0001 | <0.001 | 1.83e-08 | -1.98e-07 | 2.34e-07 | 0.868 | 0.3% | 0.794 |
| **BASnum** | .0001 | .0000 | .0001 | <0.001 | .0001 | .0000 | .0001 | <0.001 | 1.83e-07 | -4.36e-07 | 8.03e-07 | 0.562 | 3.5% | 0.001 |
| **RBC** | .0001 | .0000 | .0001 | <0.001 | .0001 | .0000 | .0001 | <0.001 | -2.71e-07 | -9.30e-07 | 3.88e-07 | 0.421 | -1.6% | 0.128 |
| **Hemoglobin** | .0001 | .0000 | .0001 | <0.001 | .0001 | .0000 | .0001 | <0.001 | 7.83e-08 | -3.90e-07 | 5.47e-07 | 0.743 | 2.9% | 0.015 |
| **Hematocrit** | .0001 | .0000 | .0001 | <0.001 | .0001 | .0000 | .0001 | <0.001 | -1.82e-10 | -3.31e-07 | 3.31e-07 | 0.999 | 4.5% | 0.245 |
| **MCV** | .0001 | .0000 | .0001 | <0.001 | .0001 | .0000 | .0001 | <0.001 | -4.06e-07 | -9.52e-07 | 1.40e-07 | 0.145 | 0.2% | 0.613 |
| **MCH** | .0001 | .0000 | .0001 | <0.001 | .0001 | .0000 | .0001 | <0.001 | -4.23e-07 | -1.10e-06 | 2.49e-07 | 0.217 | 0.8% | 0.281 |
| **MCHC** | .0001 | .0000 | .0001 | <0.001 | .0001 | .0000 | .0001 | <0.001 | 6.02e-07 | -2.41e-07 | 1.44e-06 | 0.161 | 2.8% | 0.035 |
| **RCDW** | .0001 | .0000 | .0001 | <0.001 | .0001 | .0000 | .0001 | <0.001 | 1.97e-06 | 5.79e-07 | 3.36e-06 | 0.005 | 16.4% | <0.001 |
| **PLT** | .0001 | .0000 | .0001 | <0.001 | .0001 | .0000 | .0001 | <0.001 | 4.13e-08 | -4.02e-07 | 4.84e-07 | 0.855 | -1.7% | 0.145 |
| **MPV** | .0001 | .0000 | .0001 | <0.001 | .0001 | .0000 | .0001 | <0.001 | -5.28e-08 | -5.38e-07 | 4.32e-07 | 0.831 | 0.7% | 0.442 |

**Table S7.** Overall association of physical activity and sedentary behavior with depression in survey-weighted Logistic regression models.

| **Physical activity and sitting time** | **Model 1** | **Model 2** | **Model 3** |
| --- | --- | --- | --- |
| Per-week MET of VWPA (1 SD increment) | 0.965 (0.911, 1.023) | 1.007 (0.951, 1.066) | 0.980 (0.799, 1.203) |
| Per-week MET of MWPA (1 SD increment) | 0.960 (0.904, 1.020) | 0.976 (0.919, 1.036) | 0.844 (0.655, 1.087) |
| Per-week MET of TPA (1 SD increment) | 1.009 (0.962, 1.059) | 1.015 (0.968, 1.064) | 0.853 (0.669, 1.087) |
| Per-week MET of VRPA (1 SD increment) | 0.618 (0.536, 0.714)^***^ | 0.621 (0.537, 0.718)^***^ | 0.578 (0.400, 0.836)^**^ |
| Per-week MET of MRPA (1 SD increment) | 0.764 (0.680, 0.858)^***^ | 0.776 (0.689, 0.873)^***^ | 0.524 (0.336, 0.818)^**^ |
| TMET per week (1 SD increment) | 0.907 (0.845, 0.973)^**^ | 0.943 (0.879, 1.013) | 0.827 (0.597, 1.145) |
| Sitting time (1 SD increment) | 1.100 (1.031, 1.174)^**^ | 1.115 (1.045, 1.190)^**^ | 1.370 (1.007, 1.863)^*^ |

Abbreviations: NHANES, National Health and Nutrition Examination Survey; SD, standard deviation; MET, metabolic equivalent of task; VWPA, vigorous work physical activity; MWPA, moderate work physical activity; TPA, walking or bicycling for transportation; VRPA, vigorous recreational physical activity; MRPA, moderate recreational physical activity; TMET, total MET.

Data are expressed as odds ratio (95% confidence interval).

Model 1: only survey cycle was adjusted.

Model 2: survey cycle, age, sex and race/ethnicity were adjusted.

Model 3: survey cycle, age, sex, race/ethnicity, body mass index, education, drinking, smoking, marital status, income ratio, history of diabetes, and medication for depression were adjusted.

^*^*P* < 0.05, ^**^*P* < 0.01, ^***^*P* < 0.001.

**Table S8.** Subsidiary association of physical activities and sedentary behavior with depression by covariates.

| **Subgroups** | | **Per-week MET of the following physical activity** | | | | | | |
| --- | --- | --- | --- | --- | --- | --- | --- | --- |
|  |  | **VWPA** | **MWPA** | **TPA** | **VRPA** | **MRPA** | **TMET** | **Sitting Time** |
| **Medication for Depression** | No (n=9031) | 0.951 (0.750, 1.206) | 0.821 (0.635, 1.061) | 0.846 (0.600, 1.194) | 0.589 (0.353, 0.982)^*^ | 0.433 (0.245, 0.767)^**^ | 0.830 (0.603, 1.142) | 1.381 (0.944, 2.021) |
|  | Yes (n=977) | 1.013 (0.603, 1.703) | 0.931 (0.604, 1.435) | 0.922 (0.687, 1.238) | 0.596 (0.182, 1.948) | 0.647 (0.494, 0.846)^**^ | 0.830 (0.504, 1.365) | 1.204 (0.855, 1.696) |

Abbreviations: NHANES, National Health and Nutrition Examination Survey; BMI, body mass index; MET, metabolic equivalent of task; VWPA, vigorous work physical activity; MWPA, moderate work physical activity; TPA, walking or bicycling for transportation; VRPA, vigorous recreational physical activity; MRPA, moderate recreational physical activity; TMET, total MET; NA, not available due to limited sample sizes. ^*^*P* < 0.05, ^**^*P* < 0.01, ^***^*P* < 0.001. Data are expressed as odds ratio (95% confidence interval).

**STATA code for the present analyses:**

cd "D:\ "

use "NHANES.dta", clear

drop if year == 66

rename RIDAGEYR age

rename RIAGENDR gender

rename RIDRETH1 ethnicity

rename DMDEDUC2 education

rename BMXBMI BMI

rename SMQ020 cigarettesmoking

rename ALQ101 alcoholdrinking

rename DMDMARTL maritalstatus

rename INDFMPIR income

rename DIQ010 diabetes

rename DIQ050 insulin

rename DID070 pills

rename LBXGH HbA1c

rename LBDSGLSI randomglu

rename LBDGLUSI fastingglu

rename LBDGLTSI OGTT

drop if age<18

gen agegp=.

replace agegp=1 if age>=18 & age<65

replace agegp=2 if age>=65 & age<.

label define GEN 1"male" 2"female"

label values gender GEN

label define ETH 1 "Mexican American" 2 "Other Hispanic" 3 "Non-Hispanic White" 4 "Non-Hispanic Black" 5 "Other race"

label values ethnicity ETH

recode education 7=. 9=.

label define EDU 1 "Less Than 9th Grade" 2 "9-11th Grade (Includes 12th grade with no diploma)" 3 "High School Grad/GED or Equivalent" 4 "Some College or AA degree" 5 "College Graduate or above"

label values education EDU

gen BMIgp=.

replace BMIgp=1 if BMI>0 & BMI<18.5

replace BMIgp=2 if BMI>=18.5 & BMI<25

replace BMIgp=3 if BMI>=25 & BMI<30

replace BMIgp=4 if BMI>=30 & BMI<.

recode cigarettesmoking 7=. 9=.

label define SMO 1 "Smoking" 2 "No smoking"

label values cigarettesmoking SMO

recode alcoholdrinking 7=. 9=.

label define ALD 1 "Drinking" 2 "No drinking"

label values alcoholdrinking ALD

recode maritalstatus 77=. 99=.

label define MAR 1 "Married" 2 "Widowed" 3"Divorced" 4"Separated" 5 "Never married" 6"living with partner"

label values maritalstatus MAR

gen incomegp=.

replace incomegp=1 if income>=3 & income<.

replace incomegp=2 if income>=1 & income<3

replace incomegp=3 if income<1

recode diabetes 7=. 9=. 3=. 2=0

label define DIA 1 "Yes" 2 "No"

label values diabetes DIA

recode insulin 7=. 9=. 2=0

label define INS 1 "YES" 0 "No"

label values insulin INS

recode pills 7=. 9=. 2=0

label define PIL 1 "YES" 0 "No"

label values insulin PIL

gen HbA1cgp=.

replace HbA1cgp=1 if HbA1c>6.5 & HbA1c<.

replace HbA1cgp=0 if HbA1c<=6.5

gen randomglugp=.

replace randomglugp=1 if randomglu>=11.1 & HbA1c<.

replace randomglugp=0 if randomglu<11.1

gen fastingglugp=.

replace fastingglugp=1 if fastingglu>=7.0 & HbA1c<.

replace fastingglugp=0 if fastingglu<7.0

gen OGTTgp=.

replace OGTTgp=1 if OGTT>=11.1 & OGTT<.

replace OGTTgp=0 if OGTT<11.1

gen diagnose=.

replace diagnose = 1 if diabetes ==0 & insulin==0 & pills==0

replace diagnose = 2 if diabetes ==1 | insulin ==1 | pills ==1 | HbA1cgp ==1 | randomglugp ==1 | fastingglugp ==1 | OGTTgp ==1

label define diagnose_label 1 "No" 2 "Yes"

label values diagnose diagnose_label

foreach var in DPQ010 DPQ020 DPQ030 DPQ040 DPQ050 DPQ060 DPQ070 DPQ080 DPQ090 {

recode `var' 7=. 9=.

}

drop if missing(DPQ010, DPQ020, DPQ030, DPQ040, DPQ050, DPQ060, DPQ070, DPQ080, DPQ090)

egen DPQtotal = rowtotal(DPQ010 DPQ020 DPQ030 DPQ040 DPQ050 DPQ060 DPQ070 DPQ080 DPQ090)

gen DPQtotal2=0

replace DPQtotal2=1 if DPQtotal>=10 & DPQtotal<28

rename LBXWBCSI WBC

rename LBXLYPCT LYMper

rename LBXMOPCT MONper

rename LBXNEPCT segNEUper

rename LBXEOPCT EOSper

rename LBXBAPCT BASper

rename LBDLYMNO LYMnum

rename LBDMONO MONnum

rename LBDNENO segNEUnum

rename LBDEONO EOSnum

rename LBDBANO BASnum

rename LBXRBCSI RBC

rename LBXHGB Hemoglobin

rename LBXHCT Hematocrit

rename LBXMCVSI MCV

rename LBXMCHSI MCH

rename LBXMC MCHC

rename LBXRDW RCDW

rename LBXPLTSI PLT

rename LBXMPSI MPV

rename PAQ605 vig_w

rename PAQ610 vig_wd

rename PAD615 vig_wt

rename PAQ620 mod_w

rename PAQ625 mod_wd

rename PAD630 mod_wt

rename PAQ635 walk_b

rename PAQ640 walk_bd

rename PAD645 walk_bt

rename PAQ650 vig_r

rename PAQ655 vig_rd

rename PAD660 vig_rt

rename PAQ665 mod_r

rename PAQ670 mod_rd

rename PAD675 mod_rt

recode vig_w 7=. 9=. 2=0

recode vig_wd 77=. 99=.

recode vig_wt 7777=. 9999=.

gen A = .

replace A = . if vig_w==. | vig_wd==. | vig_wt==.

replace A = 0 if vig_w==0

replace A = 8 * vig_wd * vig_wt if vig_w==1 & !missing(vig_wd, vig_wt)

recode mod_w 7=. 9=. 2=0

recode mod_wd 77=. 99=.

recode mod_wt 7777=. 9999=.

gen B = .

replace B=0 if mod_w==0

replace B =4 * mod_wd * mod_wt if mod_w==1 & !missing(mod_wd, mod_wt)

recode walk_b 7=. 9=. 2=0

recode walk_bd 77=. 99=.

recode walk_bt 7777=. 9999=.

gen C= .

replace C=0 if walk_b==0

replace C =4 * walk_bd * walk_bt if walk_b==1 & !missing(walk_bd, walk_bt)

recode vig_r 7=. 9=. 2=0

recode vig_rd 77=. 99=.

recode vig_rt 7777=. 9999=.

gen D= .

replace D=0 if vig_r==0

replace D =4 * vig_rd * vig_rt if vig_r==1 & !missing(vig_rd, vig_rt)

recode mod_r 7=. 9=. 2=0

recode mod_rd 77=. 99=.

recode mod_rt 7777=. 9999=.

gen E= .

replace E = 0 if mod_r==0

replace E = 4 * mod_rd * mod_rt if mod_r==1 & !missing(mod_rd, mod_rt)

egen total= rowtotal(A B C D E)

gen met =.

replace met = total if !missing(A, B, C, D, E)

drop if missing(met)

browse

foreach var in A B C D E met {

gen `var'2 = `var'/60

drop if missing(`var'2)

}

rename PAD680 sittingtime

recode sittingtime 7777=. 9999=.

drop if missing(sittingtime)

gen MET10R = .

replace MET10R = WTMEC2YR/7

replace SDMVPSUx = SDMVPSUy if SDMVPSUx>=.

replace SDMVSTRAx = SDMVSTRAy if SDMVSTRAx>=.

svyset SDMVPSUx [pweight = MET10R], strata(SDMVSTRAx) singleunit(centered)

foreach var in age BMI WBC segNEUnum RBC Hematocrit MCH RCDW A2 B2 C2 D2 E2 met2 sittingtime{

svy: mean `var'

svy, over(DPQtotal2): mean `var'

lincom [c.`var'@0.DPQtotal2] - [c.`var'@1.DPQtotal2]

}

foreach var of varlist age BMI WBC segNEUnum RBC Hematocrit MCH RCDW A2 B2 C2 D2 E2 met2 sittingtime {

bysort DPQtotal2: summarize `var' [aw = WTMEC2YR], detail

foreach var2 of varlist `var' {

di %9.3f `var2'

}

}

foreach var in gender ethnicity education cigarettesmoking alcoholdrinking maritalstatus incomegp diagnose{

svy: tab `var' DPQtotal2, col ci percent format(%9.3f)

}

foreach var in A2 B2 C2 D2 E2 met2 sittingtime{

egen `var'_sd = sd(`var')

gen `var'sd = `var'/`var'_sd

drop `var'_sd

}

foreach var in A2sd B2sd C2sd D2sd E2sd met2sd sittingtimesd {

svy:logistic DPQtotal2 `var' i.year

est store `var'm1

svy:logistic DPQtotal2 `var' i.year i.agegp i.gender i.ethnicity

est store `var'm2

svy:logistic DPQtotal2 `var' i.year i.agegp i.gender i.ethnicity i.BMIgp i.education i.cigarettesmoking i.alcoholdrinking i.maritalstatus i.incomegp i.diagnose

est store `var'm3

esttab `var'm1 `var'm2 `var'm3 using D:\儿研所\association\修稿\ABCDE2sdSIT.rtf, append ci eform nogap compress wide keep(`var')

}

foreach var in A2sd B2sd C2sd D2sd E2sd met2sd sittingtimesd {

svy:logistic DPQtotal2 `var' i.year i.gender i.ethnicity i.BMIgp i.education i.cigarettesmoking i.alcoholdrinking i.maritalstatus i.incomegp i.diagnose if agegp==1

est store `var'm1

svy:logistic DPQtotal2 `var' i.year i.gender i.ethnicity i.BMIgp i.education i.cigarettesmoking i.alcoholdrinking i.maritalstatus i.incomegp i.diagnose if agegp==2

est store `var'm2

svy:logistic DPQtotal2 `var' i.year i.agegp i.ethnicity i.BMIgp i.education i.cigarettesmoking i.alcoholdrinking i.maritalstatus i.incomegp i.diagnose if gender==1

est store `var'm3

svy:logistic DPQtotal2 `var' i.year i.agegp i.ethnicity i.BMIgp i.education i.cigarettesmoking i.alcoholdrinking i.maritalstatus i.incomegp i.diagnose if gender==2

est store `var'm4

svy:logistic DPQtotal2 `var' i.year i.agegp i.gender i.ethnicity i.education i.cigarettesmoking i.alcoholdrinking i.maritalstatus i.incomegp i.diagnose if BMIgp==1

est store `var'm5

svy:logistic DPQtotal2 `var' i.year i.agegp i.gender i.ethnicity i.education i.cigarettesmoking i.alcoholdrinking i.maritalstatus i.incomegp i.diagnose if BMIgp==2

est store `var'm6

svy:logistic DPQtotal2 `var' i.year i.agegp i.gender i.ethnicity i.education i.cigarettesmoking i.alcoholdrinking i.maritalstatus i.incomegp i.diagnose if BMIgp==3

est store `var'm7

svy:logistic DPQtotal2 `var' i.year i.agegp i.gender i.ethnicity i.education i.cigarettesmoking i.alcoholdrinking i.maritalstatus i.incomegp i.diagnose if BMIgp==4

est store `var'm8

svy:logistic DPQtotal2 `var' i.year i.agegp i.gender i.BMIgp i.education i.cigarettesmoking i.alcoholdrinking i.maritalstatus i.incomegp i.diagnose if ethnicity==1

est store `var'm9

svy:logistic DPQtotal2 `var' i.year i.agegp i.gender i.BMIgp i.education i.cigarettesmoking i.alcoholdrinking i.maritalstatus i.incomegp i.diagnose if ethnicity==2

est store `var'm10

svy:logistic DPQtotal2 `var' i.year i.agegp i.gender i.BMIgp i.education i.cigarettesmoking i.alcoholdrinking i.maritalstatus i.incomegp i.diagnose if ethnicity==3

est store `var'm11

svy:logistic DPQtotal2 `var' i.year i.agegp i.gender i.BMIgp i.education i.cigarettesmoking i.alcoholdrinking i.maritalstatus i.incomegp i.diagnose if ethnicity==4

est store `var'm12

svy:logistic DPQtotal2 `var' i.year i.agegp i.gender i.BMIgp i.education i.cigarettesmoking i.alcoholdrinking i.maritalstatus i.incomegp i.diagnose if ethnicity==5

est store `var'm13

svy:logistic DPQtotal2 `var' i.year i.agegp i.gender i.BMIgp i.ethnicity i.cigarettesmoking i.alcoholdrinking i.maritalstatus i.incomegp i.diagnose if education==1

est store `var'm14

svy:logistic DPQtotal2 `var' i.year i.agegp i.gender i.BMIgp i.ethnicity i.cigarettesmoking i.alcoholdrinking i.maritalstatus i.incomegp i.diagnose if education==2

est store `var'm15

svy:logistic DPQtotal2 `var' i.year i.agegp i.gender i.BMIgp i.ethnicity i.cigarettesmoking i.alcoholdrinking i.maritalstatus i.incomegp i.diagnose if education==3

est store `var'm16

svy:logistic DPQtotal2 `var' i.year i.agegp i.gender i.BMIgp i.ethnicity i.cigarettesmoking i.alcoholdrinking i.maritalstatus i.incomegp i.diagnose if education==4

est store `var'm17

svy:logistic DPQtotal2 `var' i.year i.agegp i.gender i.BMIgp i.ethnicity i.cigarettesmoking i.alcoholdrinking i.maritalstatus i.incomegp i.diagnose if education==5

est store `var'm18

svy:logistic DPQtotal2 `var' i.year i.agegp i.gender i.ethnicity i.BMIgp i.education i.alcoholdrinking i.maritalstatus i.incomegp i.diagnose if cigarettesmoking==1

est store `var'm19

svy:logistic DPQtotal2 `var' i.year i.agegp i.gender i.ethnicity i.BMIgp i.education i.alcoholdrinking i.maritalstatus i.incomegp i.diagnose if cigarettesmoking==2

est store `var'm20

svy:logistic DPQtotal2 `var' i.year i.agegp i.gender i.ethnicity i.BMIgp i.education i.cigarettesmoking i.maritalstatus i.incomegp i.diagnose if alcoholdrinking==1

est store `var'm21

svy:logistic DPQtotal2 `var' i.year i.agegp i.gender i.ethnicity i.BMIgp i.education i.cigarettesmoking i.maritalstatus i.incomegp i.diagnose if alcoholdrinking==2

est store `var'm22

svy:logistic DPQtotal2 `var' i.year i.agegp i.gender i.ethnicity i.BMIgp i.education i.cigarettesmoking i.alcoholdrinking i.incomegp i.diagnose if maritalstatus==1

est store `var'm23

svy:logistic DPQtotal2 `var' i.year i.agegp i.gender i.ethnicity i.BMIgp i.education i.cigarettesmoking i.alcoholdrinking i.incomegp i.diagnose if maritalstatus==2

est store `var'm24

svy:logistic DPQtotal2 `var' i.year i.agegp i.gender i.ethnicity i.BMIgp i.education i.cigarettesmoking i.alcoholdrinking i.incomegp i.diagnose if maritalstatus==3

est store `var'm25

svy:logistic DPQtotal2 `var' i.year i.agegp i.gender i.ethnicity i.BMIgp i.education i.cigarettesmoking i.alcoholdrinking i.incomegp i.diagnose if maritalstatus==4

est store `var'm26

svy:logistic DPQtotal2 `var' i.year i.agegp i.gender i.ethnicity i.BMIgp i.education i.cigarettesmoking i.alcoholdrinking i.incomegp i.diagnose if maritalstatus==5

est store `var'm27

svy:logistic DPQtotal2 `var' i.year i.agegp i.gender i.ethnicity i.BMIgp i.education i.cigarettesmoking i.alcoholdrinking i.incomegp i.diagnose if maritalstatus==6

est store `var'm28

svy:logistic DPQtotal2 `var' i.year i.agegp i.gender i.ethnicity i.BMIgp i.education i.cigarettesmoking i.alcoholdrinking i.maritalstatus i.diagnose if incomegp==1

est store `var'm29

svy:logistic DPQtotal2 `var' i.year i.agegp i.gender i.ethnicity i.BMIgp i.education i.cigarettesmoking i.alcoholdrinking i.maritalstatus i.diagnose if incomegp==2

est store `var'm30

svy:logistic DPQtotal2 `var' i.year i.agegp i.gender i.ethnicity i.BMIgp i.education i.cigarettesmoking i.alcoholdrinking i.maritalstatus i.diagnose if incomegp==3

est store `var'm31

svy:logistic DPQtotal2 `var' i.year i.agegp i.gender i.ethnicity i.BMIgp i.education i.cigarettesmoking i.alcoholdrinking i.maritalstatus i.incomegp if diagnose==1

est store `var'm32

svy:logistic DPQtotal2 `var' i.year i.agegp i.gender i.ethnicity i.BMIgp i.education i.cigarettesmoking i.alcoholdrinking i.maritalstatus i.incomegp if diagnose==2

est store `var'm33

esttab `var'm1 `var'm2 `var'm3 `var'm4 `var'm5 `var'm6 `var'm7 `var'm8 `var'm9 `var'm10 `var'm11 `var'm12 `var'm13 `var'm14 `var'm15 `var'm16 `var'm17 `var'm18 `var'm19 `var'm20 `var'm21 `var'm22 `var'm23 `var'm24 `var'm25 `var'm26 `var'm27 `var'm28 `var'm29 `var'm30 `var'm31 `var'm32 `var'm33 using D:\儿研所\association\修稿\subgp_ABCDE2sdSIT.rtf, append ci eform nogap compress wide keep(`var')

}

foreach var in year agegp gender ethnicity BMIgp education cigarettesmoking alcoholdrinking maritalstatus incomegp diagnose {

gen `var'_interA2sd=.

replace `var'_interA2sd=`var' * A2sd if A2sd > 0 & A2sd < . & `var' > 0 & `var' < .

svy: logistic DPQtotal2 A2sd `var_interA2sd' year agegp gender ethnicity BMIgp education cigarettesmoking alcoholdrinking maritalstatus incomegp diagnose

}

foreach var in year agegp gender ethnicity BMIgp education cigarettesmoking alcoholdrinking maritalstatus incomegp diagnose {

gen `var'_interB2sd=.

replace `var'_interB2sd=`var' * B2sd if B2sd > 0 & B2sd < . & `var' > 0 & `var' < .

svy: logistic DPQtotal2 B2sd `var_interB2sd' year agegp gender ethnicity BMIgp education cigarettesmoking alcoholdrinking maritalstatus incomegp diagnose

}

foreach var in year agegp gender ethnicity BMIgp education cigarettesmoking alcoholdrinking maritalstatus incomegp diagnose {

gen `var'_interC2sd=.

replace `var'_interC2sd=`var' * C2sd if C2sd > 0 & C2sd < . & `var' > 0 & `var' < .

svy: logistic DPQtotal2 C2sd `var_interC2sd' year agegp gender ethnicity BMIgp education cigarettesmoking alcoholdrinking maritalstatus incomegp diagnose

}

foreach var in year agegp gender ethnicity BMIgp education cigarettesmoking alcoholdrinking maritalstatus incomegp diagnose {

gen `var'_interD2sd=.

replace `var'_interD2sd=`var' * D2sd if D2sd > 0 & D2sd < . & `var' > 0 & `var' < .

svy: logistic DPQtotal2 D2sd `var_interD2sd' year agegp gender ethnicity BMIgp education cigarettesmoking alcoholdrinking maritalstatus incomegp diagnose

}

foreach var in year agegp gender ethnicity BMIgp education cigarettesmoking alcoholdrinking maritalstatus incomegp diagnose {

gen `var'_interE2sd=.

replace `var'_interE2sd=`var' * E2sd if E2sd > 0 & E2sd < . & `var' > 0 & `var' < .

svy: logistic DPQtotal2 E2sd `var_interE2sd' year agegp gender ethnicity BMIgp education cigarettesmoking alcoholdrinking maritalstatus incomegp diagnose

}

foreach var in year agegp gender ethnicity BMIgp education cigarettesmoking alcoholdrinking maritalstatus incomegp diagnose {

gen `var'_intermet2sd=.

replace `var'_intermet2sd=`var' * met2sd if met2sd > 0 & met2sd < . & `var' > 0 & `var' < .

svy: logistic DPQtotal2 met2sd `var_intermet2sd' year agegp gender ethnicity BMIgp education cigarettesmoking alcoholdrinking maritalstatus incomegp diagnose

}

foreach var in year agegp gender ethnicity BMIgp education cigarettesmoking alcoholdrinking maritalstatus incomegp diagnose {

gen `var'_intersit=.

replace `var'_intersit=`var' * sittingtimesd if sittingtimesd > 0 & sittingtimesd < . & `var' > 0 & `var' < .

svy: logistic DPQtotal2 sittingtimesd `var_intersit' year agegp gender ethnicity BMIgp education cigarettesmoking alcoholdrinking maritalstatus incomegp diagnose

}

log using mediation3, t

foreach var in WBC LYMper MONper segNEUper EOSper BASper LYMnum MONnum segNEUnum EOSnum BASnum RBC Hemoglobin Hematocrit MCV MCH MCHC RCDW PLT MPV {

foreach var2 in A2 B2 D2 E2 met2 sittingtime {

sgmediation2 DPQtotal2, mv(`var') iv(`var2')

bootstrap r(ind_eff) r(dir_eff) r(tot_eff), reps(50): sgmediation2 DPQtotal2, mv(`var') iv(`var2') cv(i.year i.agegp i.gender i.BMIgp i.ethnicity i.education i.cigarettesmoking i.alcoholdrinking i.maritalstatus i.incomegp i.diagnose)

}

}

foreach var in DPQtotal2{

di "`var': 0"

sktest age BMI WBC segNEUnum RBC Hematocrit MCH RCDW A B C D E met sittingtime if `var'==0

di "`var': 1"

sktest age BMI WBC segNEUnum RBC Hematocrit MCH RCDW A B C D E met sittingtime if `var'==1

}

foreach var in age BMI CRP WBC segNEUnum RBC Hematocrit MCH RCDW NLR A B C D E met sittingtime{

kwallis `var', by(DPQtotal2)

di "p value for `var': " 2*(1-normal(abs(r(z))))

}

set matsize 5000

outreg2 using table1.doc,replace sum(detail) keep(age BMI CRP WBC segNEUnum RBC Hematocrit MCH RCDW NLR A2 B2 C2 D2 E2 met2 sittingtime) eqkeep(N mean sd p25 p75) dec(2)

bysort DPQtotal2: outreg2 using with&without.doc, replace sum(detail) keep(age BMI CRP WBC segNEUnum RBC Hematocrit MCH RCDW NLR A2 B2 C2 D2 E2 met2 sittingtime) eqkeep(p50 p25 p75) dec(2)

foreach var in gender ethnicity education cigarettesmoking alcoholdrinking aritalstatus incomegp diagnose {

tabout `var' DPQtotal2 using table1_2.xls, percent c(freq col) stats(chi2) append

}
